# Edaravone and mitochondrial transfer as potential therapeutics for vanishing white matter disease astrocyte dysfunction

**DOI:** 10.1111/cns.14190

**Published:** 2023-03-27

**Authors:** Neville S. Ng, Michelle Newbery, Aude Touffu, Simon Maksour, Johnson Chung, Luke Carroll, Thiri Zaw, Yunqi Wu, Lezanne Ooi

**Affiliations:** ^1^ Illawarra Health and Medical Research Institute Wollongong New South Wales Australia; ^2^ School of Chemistry and Molecular Bioscience and Molecular Horizons University of Wollongong Wollongong New South Wales Australia; ^3^ Intelligent Polymer Research Institute, ARC Centre of Excellence for Electromaterials Science, AIIM Facility, Innovation Campus University of Wollongong Wollongong New South Wales Australia; ^4^ Australian Proteome Analysis Facility (APAF) Macquarie University Sydney New South Wales Australia

**Keywords:** astrocyte, edaravone, induced pluripotent stem cells, mitochondria, mitochondrial transfer, vanishing white matter disease

## Abstract

**Introduction:**

Previous research has suggested that vanishing white matter disease (VWMD) astrocytes fail to fully differentiate and respond differently to cellular stresses compared to healthy astrocytes. However, few studies have investigated potential VWMD therapeutics in monoculture patient‐derived cell‐based models.

**Methods:**

To investigate the impact of alterations in astrocyte expression and function in VWMD, astrocytes were differentiated from patient and control induced pluripotent stem cells and analyzed by proteomics, pathway analysis, and functional assays, in the absence and presence of stressors or potential therapeutics.

**Results:**

Vanishing white matter disease astrocytes demonstrated significantly reduced expression of astrocyte markers and markers of inflammatory activation or cellular stress relative to control astrocytes. These alterations were identified both in the presence and absence of polyinosinic:polycytidylic acid stimuli, which is used to simulate viral infections. Pathway analysis highlighted differential signaling in multiple pathways in VWMD astrocytes, including eukaryotic initiation factor 2 (EIF2) signaling, oxidative stress, oxidative phosphorylation (OXPHOS), mitochondrial function, the unfolded protein response (UPR), phagosome regulation, autophagy, ER stress, tricarboxylic acid cycle (TCA) cycle, glycolysis, tRNA signaling, and senescence pathways. Since oxidative stress and mitochondrial function were two of the key pathways affected, we investigated whether two independent therapeutic strategies could ameliorate astrocyte dysfunction: edaravone treatment and mitochondrial transfer. Edaravone treatment reduced differential VWMD protein expression of the UPR, phagosome regulation, ubiquitination, autophagy, ER stress, senescence, and TCA cycle pathways. Meanwhile, mitochondrial transfer decreased VWMD differential expression of the UPR, glycolysis, calcium transport, phagosome formation, and ER stress pathways, while further modulating EIF2 signaling, tRNA signaling, TCA cycle, and OXPHOS pathways. Mitochondrial transfer also increased the gene and protein expression of the astrocyte marker, glial fibrillary acidic protein (GFAP) in VWMD astrocytes.

**Conclusion:**

This study provides further insight into the etiology of VWMD astrocytic failure and suggests edaravone and mitochondrial transfer as potential candidate VWMD therapeutics that can ameliorate disease pathways in astrocytes related to oxidative stress, mitochondrial dysfunction, and proteostasis.

## Graphical abstract

Induced pluripotent stem cell‐derived astrocytes from vanishing white matter disease (VWMD) patients demonstrate aberrant mitochondrial membrane potential (ΔΨm), changes in proteins important for glycolysis, the cytoskeleton and motility signaling, disruption to the unfolded protein response and increased oxidative stress. Since mitochondrial function and oxidative stress appeared as significant VWMD cellular phenotypes, two therapeutic approaches were trialed in VWMD astrocytes in vitro. Edaravone, a drug currently used for amyotrophic lateral sclerosis, reverted the VWMD phenotype in cells, decreasing oxidative stress, disruption to the unfolded protein response and protein ubiquitination, and upregulating oxidative phosphorylation pathways. Mitochondrial transfer improved the phenotype of VWMD cells, increasing mitochondrial membrane potential, reverting the disruption to the UPR, decreasing the proteomic phenotype associated with aberrant glycolysis and cytoskeletal and motility signaling and upregulating oxidative phosphorylation. This work suggests that the deficits in mitochondrial function, increased oxidative stress and structural protein expression in VWMD can be targeted to revert these phenotypes.

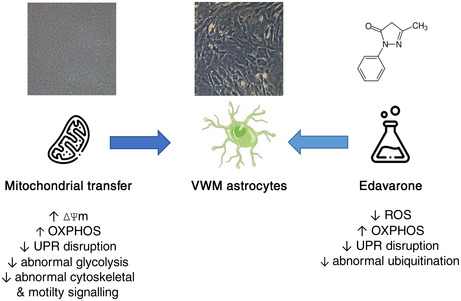

## 1 INTRODUCTION

Childhood ataxia with central nervous system hypomyelination (CACH) or vanishing white matter disease (VWMD) is a chronic progressive neurological disease that in prenatal and congenital forms presents as severe encephalopathy and rapid decline following head trauma,^1^ major surgery, infections, or acute and extreme fright.^2^ VWMD is caused by mutations in the genes, *EIF2B1, EIF2B2, EIF2B3 EIF2B4*, and *EIF2B5*, encoding the five subunits of the eIF2B protein (several hundred missense mutations across the subunits), which impacts global mRNA translation. Although eIF2B is expressed in all cells, the major symptoms are attributable to demyelination, due to dysfunctional oligodendrocytes and astrocytes. The process of myelination is facilitated primarily by oligodendrocyte maturation and function, but is also highly dependent on the support of astrocyte secretion by platelet‐derived growth factor‐α, fatty acids, lipids,^3^ and insulin‐like growth factor 1.^4^, ^5^

Astrocytes release cytokines and extracellular matrix remodeling molecules that aid plasticity during neural development, as well as myelination inhibiting, myelin degrading, or pro‐myelination factors while switching between reactive and non‐reactive states with distinct gene expression profiles and morphologies.^6^, ^7^, ^8^ Astrocytes have been identified as a critical component of oligodendrocyte precursor cell (OPC) survival, maturation, and function in VWMD dysfunction.^9^ Conditioned media from VWMD induced pluripotent stem cell (iPSC)‐derived astrocytes has been demonstrated to inhibit OPC maturation as a result of hyaluronic acid, which was reversed by the presence of hyaluronidase.^9^ Meanwhile, primary mouse VWMD *Eif2b* ^R132H^/^R132H^ astrocytes have been observed to show upregulated oxidative stress and mitochondrial transcript levels, as well as proteasomal inhibition,^10^ which can inhibit the autophagy‐lysosomal pathway.^11^

Although patient and animal model studies provide a valuable source of pathophysiology data, monoculture stem cell models provide an accessible route for modeling human patient cell pathology. At present, studies exploring VWMD therapeutics have included guanabenz,^12^ ISRIB,^13^ 2BAct,^14^ and sigma‐1 agonists.^15^ We recently identified edaravone, an antioxidant and anti‐inflammatory^16^ medication utilized for acute ischemic stroke in Japan^17^ and amyotrophic lateral sclerosis (ALS) in USA and Japan,^18^ as a cytoprotective‐free radical scavenger that may ameliorate the cytotoxic effects of endoplasmic reticulum (ER) stress in VWMD astrocytes,^19^ although further studies are required to investigate its mechanism of action. At least some of the cell physiological defects in VWMD astrocytes are associated with mitochondrial dysfunction, however, no reported studies have investigated mitochondrial transfer as a potential VWMD therapeutic. In this study, we investigated the stress responses and mitochondrial VWMD disease phenotype of patient iPSC‐derived astrocytes, and edaravone and mitochondrial transfer as potential therapeutics.

## 2 METHODS

### 2.1 Cell culture and differentiations

The iPSCs derived from human patient fibroblasts were previously reprogrammed by mRNA overexpression of Oct4, Sox2, Klf4, c‐Myc, Nanog, and Lin28 as previously published.^19^ All vessels were coated with Matrigel (BDAA354277, Bio‐Strategy). Cells were incubated with DMEM/F12, 2% B‐27 (17504001, Thermo Fisher Scientific), and GlutaMAX (35050061) with 0.1 μM LDN193189 (HY‐12071A‐10MG, Focus Bioscience) and 10 μM SB431542 (HY‐10431‐10MG, Focus Bioscience) for 5 days. The small molecules were then replaced with 20 ng/mL EGF (130‐097‐749, Miltentyi Biotec GmbH) and 20 ng/mL FGF‐2 (130‐093‐841, Miltentyi Biotec GmbH) for 5 days. Astrocytes were differentiated by transduction of neural precursor cells (NPCs) with lentiviral particles carrying SRY‐Box Transcription Factor 9 (SOX9) and Nuclear Factor I B (NFIB), based on an adaptation of a previously described protocol.^20^ Briefly, NPCs were transduced with lentiviral viral particles overnight before incubation with 2 μg/mL doxycycline (D3447‐500MG, Sigma‐Aldrich) for 10 days, followed by selection with 2 μg/mL puromycin (P8833‐10MG, Sigma‐Aldrich) for 3 days. Astrocytes were cultured in DMEM/F12, 2 mM GlutaMAX, 2% B‐27, 10 ng/mL CNTF, BMP4, FGF2, EGF, and 250 μM dbCAMP (D0627‐1G, Sigma‐Aldrich) for characterization at day 14–16, and utilized for downstream experiments until day 42. Microplate cell culture plate seeding and assay reagent handling were performed with a Hamilton Microlab STAR automated liquid handler and custom user interface.^21^

### 2.2 Mitochondrial transfer

Live mitochondria were extracted using an adaptation of previously utilized methods as optimized with a combined Dounce and needle homogenization method (Figure S7). Briefly, cells were resuspended in extraction buffer (320 mM sucrose, 1 mM EGTA, 10 mM KCl, 0.1% BSA, 10 mM HEPES, pH 7.4), and the pellet subjected to Dounce homogenization and trituration with a 25G needle. Cell debris was removed by two centrifugations at 1500x ***g*** for 3 min. Mitochondria were isolated by centrifugation of cell extract at 5500x ***g*** for 10 min and resuspended in an extraction buffer. Electroporation experiments were performed with a Bio‐Rad Gene Pulser at field strengths 1.25–12.5 kV/cm, 25 microfarad capacitance, 400 Ohms resistance, and mitochondria resuspended in extraction buffer with 50 ng/μL plasmid, resuspended in extraction buffer, similar to as previously described.^22^ Cell cultures were centrifuged with mitochondrial extract at 1500x ***g*** for 15 min at 4°C and coincubated with 1.5 mM EGTA similar to as previously described.^23^

### 2.3 Dichlorodihydrofluorescein (DCF) assay

Cells were seeded at a density of 10^4^ per well in 96 well plates overnight and incubated with cell‐permeant 1 μM 2′,7′‐dichlorodihydrofluorescein diacetate (H2DCFDA; D399, Thermo Fisher Scientific). Microplates were analyzed with a BMG Polarstar fluorescence spectrometer (ex 485‐12/em 520). Cell cultures were normalized to cellular protein as previously described.^24^ Cell cultures were fixed with cold 10% (w/v) trichloroacetic acid (T4885‐1KG, Sigma‐Aldrich) and 0.004% (w/v) Sulforhodamine B (230162‐5G, Sigma‐Aldrich) solution, washed twice with 1% (w/v) acetic acid (A6283, Sigma‐Aldrich) and analyzed by fluorescence spectrometry (ex 544/em 590).

### 2.4 Mitochondrial membrane potential (ΔΨm) assay

Cells were seeded at a density of 10^4^ per well in 96 well plates overnight and incubated with 0.2 μM Tetramethylrhodamine Ethyl Ester (TMRE; T669, Thermo Fisher Scientific) before imaging with an Incucyte S3 Live Analysis System (Sartorius).

### 2.5 Resazurin reduction assay

Cell cultures were seeded as described above and incubated with 15 μM resazurin for 0.5 h before analysis by fluorescence spectrometry (ex 544/em 590), and normalization to cellular protein measured by a Pierce BCA Protein Assay Kit (23225, Thermo Fisher Scientific).

### 2.6 ATP assay

Assay reagents were prepared according to manufacturer instructions (A22066, Thermo Fisher Scientific). Briefly, the reaction solution was diluted 20×, before the addition of DTT, luciferin, and luciferase to a final composition of 0.5 mM D‐luciferin, 1.25 μg/mL firefly luciferase, 25 mM Tricine buffer, pH 7.8, 5 mM MgSO4, 100 μM EDTA and 1 mM DTT. Cells were seeded at a density of 10^4^ per well in 96 well plates and were lysed in TBST, before the addition of 5 μL of cell lysate to 50 μL of the standard reaction solution, with the luminescence assay performed with duplicate wells. Samples were normalized to cellular protein measured by BCA assay.

### 2.7 Glutamate assay

Intracellular glutamate was measured using the manufacturer's protocol (ab138883, Abcam). Astrocytes at a density of 10^4^ per well were seeded in 96‐well plates and lysed in TBST before the addition of 50 μL lysis sample to 50 μL reaction mix and incubation for 0.5 h. Microplates were analyzed by fluorescence spectroscopy (ex 540/em 590) and data normalized to cellular protein was measured by BCA assay.

### 2.8 Quantitative PCR (qPCR)

Real‐time qPCR was performed as per manufacturer settings with TaqMan Fast Advanced Master Mix (4444556, Thermo Fisher Scientific), and predesigned assays Hs02596860_s1 (*MT‐RNR2*) and Hs02758991_g1 (*GAPDH*). Thermocycling was performed with Uracil‐DNA‐glycosylase activation at 50°C for 2 min, polymerase activation at 95°C for 20 s, and 40 cycles of elongation with denaturing at 95°C for 1 s and annealing at 60°C 20 s. Reactions were performed in duplicate with an Applied Biosystems Scientific QuantStudio 5, and data were processed with Quantstudio 5.

### 2.9 Reverse Transcription Quantitative PCR (RT‐qPCR)

Gene expression assays were performed with a direct lysis microplate assay.^25^ Cells were washed with 0.9% w/v saline before lysis in 5 mM Tris, 75 mM saline, and 0.05% Triton X‐100 for 5 min. RT‐qPCR reactions were performed with MicroAmp 96‐Well Reaction Plates (4366932, Thermo Fisher Scientific). Thermal cycling was performed with a 5 μL template and 5 μL reverse transcriptase TaqPath 1‐Step RT‐qPCR Master Mix (A15300, Thermo Fisher Scientific) at 50°C for 15 min, enzyme activation 2 min, amplification 95°C for 3 s/60°C for 15 s (Applied Biosystems QuantStudio 5). TaqMan predesigned assays (exon junction spanning) were used, including Hs00389217_m1 (*S100B*), Hs00188193_m1 (*SLC1A3*), Hs01102423_m1 (*SLC1A2*), Hs00909233_m1 (*GFAP*), Hs02596860_s1 (*MT‐RNR2*), Hs00173304_m1 (*PPARGC1A*), Hs01009006_m1 (*SIRT1*), Hs02758991_g1 (*GAPDH*), and Hs00427620_m1 (*TBP*) (4,331,182, Thermo Fisher Scientific). For *IL6* activation assays, astrocyte cultures were treated with saline or 100 μg/mL polyinosinic:polycytidylic acid (poly(I:C)) overnight. Data were acquired with an Applied Biosystems Scientific QuantStudio 5 and analyzed with Quantstudio v1.5.1.

### 2.10 Immunofluorescence

Cells were seeded at a density of 5000 per well in Matrigel‐coated 96‐well plates overnight. Samples were fixed in 4% paraformaldehyde for 10 min, before blocking for 10 min in 5% donkey serum (S30‐100ML, Merck), and staining with primary antibody in 1% donkey serum overnight at 4 °C (1:300 ab76997 Anti‐SOX9 antibody mouse monoclonal, ab186738 Anti‐NFIB antibody rabbit monoclonal, ab68428 Anti‐GFAP antibody rabbit monoclonal, ab416 Anti‐EAAT1 rabbit polyclonal, ab190368 Anti‐ALDOC mouse monoclonal, MA1‐110, Thermo Fisher Scientific Anti‐Nestin mouse monoclonal). Cells were washed three times in TBST for 5 min before staining with secondary antibody and 10 μM Hoechst 33342 for 1 h (1:500, ab150105, Alexa Fluor 488 Donkey Anti‐Mouse IgG H&L, ab150075, Alexa Fluor 647 Donkey Anti‐Rabbit IgG H&L and washed three times in TBST). Images were acquired with a Leica SP8 confocal.

### 2.11 Western blot

Fluorescent western blots were performed as previously published (19). Samples lysed in RIPA buffer (50 mM Tris, 150 mM NaCl, 1% Triton X‐100, 0.5% (w/v) sodium deoxycholate, 0.1% (w/v) SDS) were loaded in Laemmli buffer in 5% β‐mercaptoethanol and separated by electrophoresis with Bio‐Rad Criterion TGX Stain‐Free Precast Gel (4%–20%; Bio‐rad, 5,678,095) in a Bio‐Rad Criterion Cell, before transfer with a Criterion Blotter to Immobilon‐FL PVDF 0.45 um (Merck, IPFL00010) membrane. Immunoblots were blocked with Blocker FL Fluorescent Blocking Buffer (37565, Thermo Fisher), incubated with primary anti‐GFAP rabbit antibody (AB5804, Sigma‐Aldrich), for 16 h at 4°C (1:2000), and secondary anti‐rabbit Alexa Fluor Plus 647 antibody (A32795, Thermo Fisher) for 1 h (1:5000), facilitated with an automated staining system GOBlot (Cytoskeleton). Fluorescent immunoblots were visualized with a ChemiDoc MP (Bio‐Rad; Figure S10).

### 2.12 Proteomics: Sample preparation

Samples were lysed by the addition of 1% sodium deoxycholate in 50 mM triethyl ammonium bicarbonate (pH 8.5) and tip‐probe sonicated. Aliquots of 50 μg protein were taken and reduced with dithiothreitol (10 mM; 60°C for 30 min) and alkylated with iodoacetamide (20 mM; ambient temperature in dark for 30 min), followed by digestion with trypsin for 16 h at 37°C. sodium deoxycholate was removed by trifluoroacetic acid precipitation followed by desalting using a solid phase extraction disk Styrene Divinyl Benzene containing Stage tips (Empore SDB‐RPS 47 mm extraction disk, SUPLCO), as previously described (https://www.nature.com/articles/nprot.2007.261). Samples were reconstituted in 50 μL 0.2% formic acid for liquid chromatography–mass spectrometry (LC–MS) analysis.

A pooled sample was generated by mixing 12 μg aliquots of each digested peptide sample for fractionation by high pH reverse phase LC, dried and resuspended at 1 μL μg^−1^ in 5 mM ammonium hydroxide solution (pH 10.5). An aliquot of 300 μg of the pooled sample was injected onto a Zorbax 300Extend‐C18 3.5 μm, 2.1 × 150 mm column (Agilent) using and Agilent 1260 Ultra‐High‐Performance Liquid Chromatography (UHPLC) system, and peptides separated by gradient elution with buffer A (5 mM ammonium hydroxide solution (pH 10.5) and buffer B consisting of 5 mM ammonia solution in 90% acetonitrile (pH 10.5)), with a flow rate of 0.3 mL min^−1^ and initial concentration of 3% B. Buffer B was held at 3% for 10 min before increasing to 30% over 55 min and then to 70% between 55 and 65 min and 90% between 65 and 70 min. The eluent was collected every 2 min for the first 10 min and at 1 min intervals for the rest of the gradient. Seventeen fractions were concatenated (0–85 min), dried and resuspended in 31 μL of loading buffer. Each fraction (10 μL) was analyzed by LC–MS by information‐dependent acquisition (2D‐IDA) analysis.

### 2.13 Proteomics: LC–MS acquisition

Peptides were separated and analyzed by Liquid Chromatography‐Electrospray Ionization‐Mass Spectrometry (LC‐ESI MS/MS) Triple TOF 6600 (Sciex) coupled to an Eksigent Ultra nanoLC system (Eksigent).^26^ Samples and high pH fraction were loaded (10 μL) onto a peptide trap (5 mm × 300 μm, μ‐Precolumn C18 PepMep 100, 5 μm, 100 Å (ThermoFisher Scientific)) at 5 μL for 3 min before the gradient was switched in‐line with an in‐house packed column (20 cm × 200 μm, Solidcore Halo 2.7 μm 160 Å ES‐C18). Peptides were eluted from the column using a linear solvent gradient with mobile phase A consisting of 0.1% formic acid and mobile phase B consisting of 99.9% acetonitrile, 0.1% formic acid with a flow rate of 600 μL min^−1^. B was initially set to 5% before increasing to 35% over 60 min for peptide elution, and the column was cleaned with 95% B for 6 min and then equilibrated with 5% B for 10 min before the next sample injection. Reverse phase eluent was subject to positive ion nano‐flow electrospray analysis, fractions were analyzed by IDA methods to generate ion libraries, and quantitative samples were analyzed by sequential window acquisition of all theoretical mass spectra (SWATH‐MS) method. In the IDA method, a survey scan was acquired (*m/z* 350–1500, 0.25 s) with the 20 most intense multiply charged ions (2^+^ − 5^+^; exceeding 200 counts per s) in the scan sequentially subjected to tandem mass spectrometry (MS/MS) analysis. MS/MS spectra were accumulated for 100 ms in the mass range *m/z* 100–1800 with rolling collision energy. In the SWATH method, a TOF‐MS survey scan was acquired (*m/z* 350–1500, 50 ms) then the 100 predefined *m/z* ranges were sequentially subjected to MS/MS analysis. MS/MS spectra were accumulated for 30 ms in the mass range *m/z* 350–1500.

The data files generated by IDA analysis were searched with ProteinPilot (v5.0; Sciex) using the ParagonTM algorithm in thorough mode. Homo sapiens protein database (reviewed) was downloaded from Uniprot (accessed May 2021, containing 26,579 proteins) and used for searching the data, with carbamidomethylation of Cys residues selected as a fixed modification. An Unused Score cut‐off was set to 1.3 (95% confidence for identification), and a global protein false discovery rate (FDR) of 1%. The top 6 most intense fragments of each peptide were extracted from the SWATH data (75 ppm mass tolerance, 5 min retention time window) based on a local ion library containing 2920 proteins constructed from IDA runs in PeakView (v2.2, Sciex), with shared and modified peptides excluded. After data processing, peptides (max 100 peptides per protein) with confidence 99% and FDR 1% (based on chromatographic feature after fragment extraction) were used for quantitation. The protein peak areas were normalized to the total peak area of the respective sample and relative protein peak areas between the sample groups compared by *t*‐test in R. Differentially expressed proteins were identified by a *t*‐test with a *p*‐value smaller than 0.05 and a minimum fold‐change of 1.5. Canonical pathway analysis was conducted with significant differentially expressed proteins identified from SWATH‐MS in QIAGEN Ingenuity Pathway Analysis (IPA) software.

### 2.14 Statistics

Data were analyzed by two‐tailed Student's *t*‐tests or one‐way analysis of variance (ANOVA) with Holm‐Sidak post hoc multiple comparisons test. The Shapiro–Wilk test was performed for the evaluation of normality.

## 3 RESULTS AND DISCUSSION

### 3.1 Altered gene expression and function of VWMD astrocytes

**FIGURE 1:**
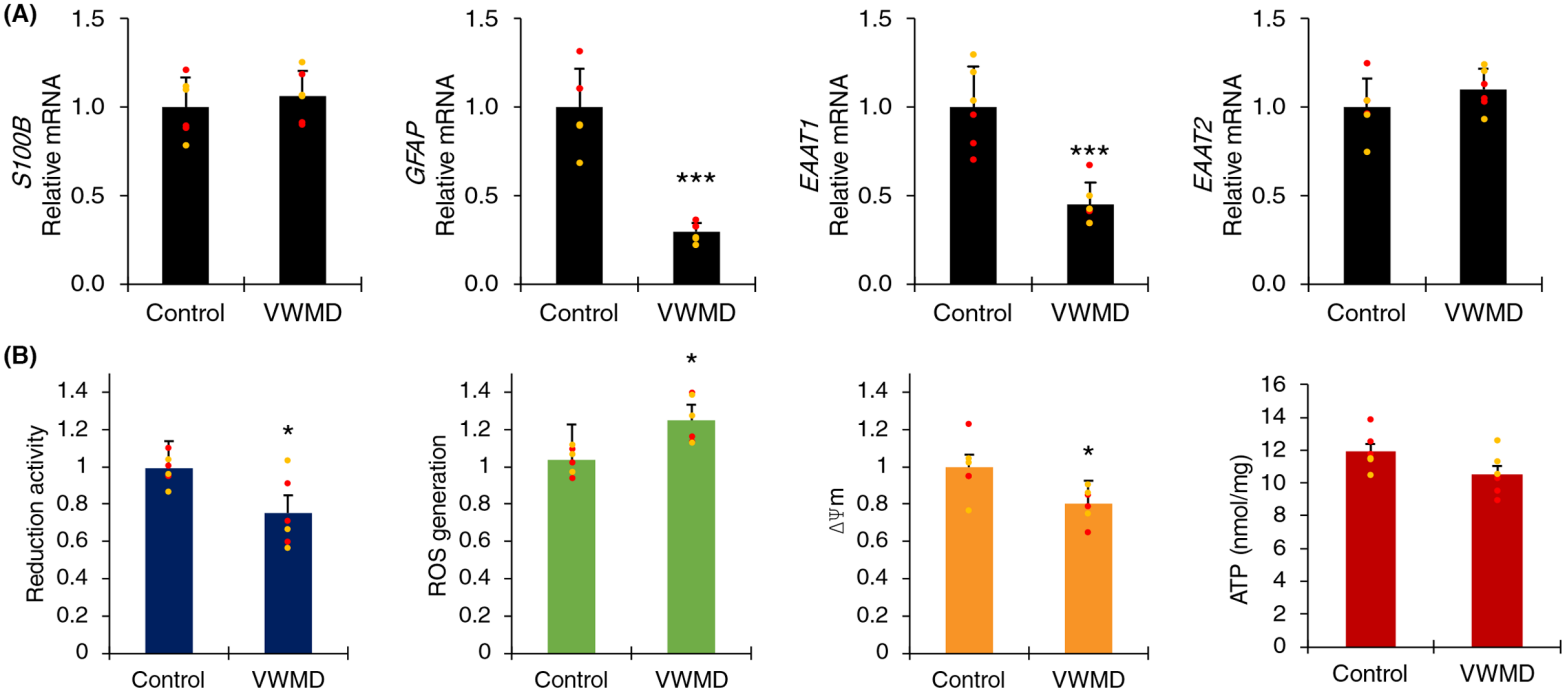
Vanishing white matter disease (VWMD) astrocyte gene expression, reduction activity, oxidative stress (reactive oxygen species (ROS) generation), mitochondrial membrane potential (ΔΨm), and ATP assay data. Data are presented as mean ± SEM (*n* = 3), *** *p* < 0.001 disease group compared to control (red = *EIF2B5* VWMD and control and orange *= EIF2B2* VWMD and control lines) Student's *t*‐test.

Vanishing white matter disease astrocytes were generated using an adaptation of a previously published protocol (Figure S1).^20^, ^25^ Gene expression characterization indicated significantly decreased *GFAP* and *EAAT1* gene expression in VWMD astrocytes compared to control astrocytes (Figure 1A).^20^ VWMD astrocytes also showed decreased reduction activity, increased reactive oxygen species, and a minor decrease in mitochondrial membrane potential (ΔΨm) (Figure 1B). No significant difference in intracellular ATP levels was detected. No change in protein synthesis rate was observed between VWMD astrocytes compared to control astrocytes (Figure S2).

The role of eIF2B on metabolic and redox homeostasis in astrocytes has been previously investigated in primary mouse VWMD *Eif2b5* astrocytes, which have been observed to show upregulated oxidative stress and mitochondrial transcriptional levels, as well as proteasomal inhibition,^10^ and decreased intracellular ATP.^27^ The lack of difference in homopropargylglycine (HPG)‐tagged nascent protein synthesis in iPSC‐derived astrocytes under basal and stressed conditions were similar to prior studies involving knock‐in mutations,^13^ suggesting that the VWMD disease mechanism is more complex than a simple change in protein synthesis rate.

**FIGURE 2:**
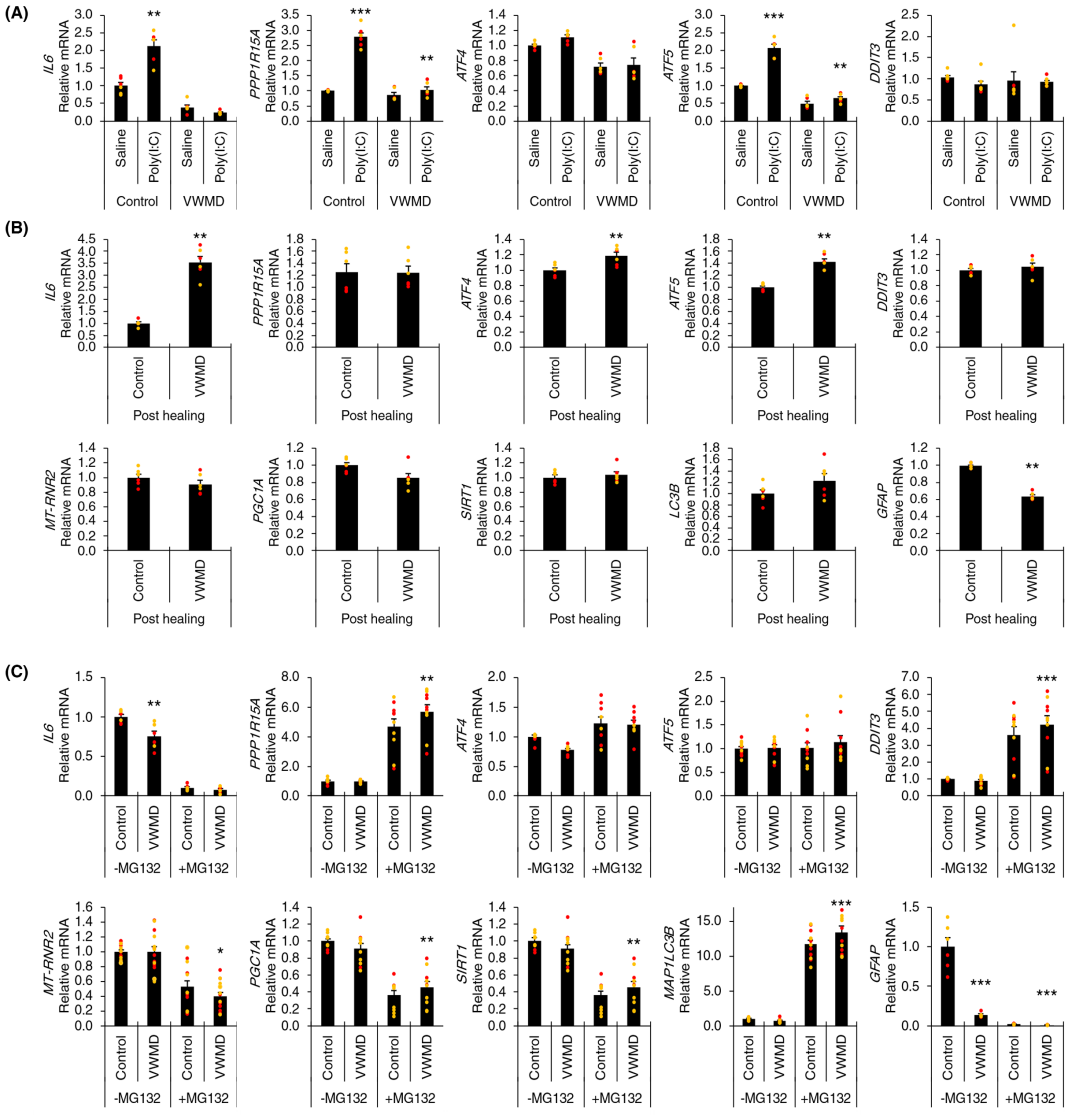
Differential response of vanishing white matter disease (VWMD) astrocytes to poly(I:C) and MG132‐stressed conditions. (A) Gene expression of inflammatory (*IL6*) and integrated stress response (ISR) (*PPP1R15A*, *ATF4*, *ATF5*, *DDIT3*) markers in VWMD astrocytes in response to poly(I:C) compared to untreated group. (B) Post‐healing differential gene expression following scratch wound assay. (C) Gene expression of mitochondrial copy (*MT‐RNR2*), mitochondrial biogenesis (*PGC1A*, *SIRT1*), autophagy (*MAP1LC3B*), and astrocyte reactivity markers (GFAP) in the presence and absence of 0.1 μM MG132 for 20 h. ** *p* < 0.01, *** *p* < 0.001 disease group compared to control. Data presented as mean ± SEM (*n* = 3, red = *EIF2B5* disease and control, and orange *= EIF2B2* disease and control; one‐way ANOVA with Holm‐Sidak post hoc multiple comparisons test).

Vanishing white matter disease is caused by mutations in genes encoding eIF2B protein subunits that are central to translation regulation and the integrated stress response. In patients, the condition is worsened following viral infections. Consequently, the expression of genes relevant to inflammatory signaling and the integrated stress response in VWMD astrocytes was investigated. Polyinosinic:polycytidylic acid (poly(I:C)) is an immunostimulant that is used to mimic viral infection in vitro and in vivo. To investigate the response to poly(I:C), gene expression of a range of inflammatory and cell stress markers was assessed in VWMD astrocytes compared to control astrocytes. While *IL6* (encodes interleukin‐6, a pro‐inflammatory cytokine in astrocytes), *PPP1R15A* (encodes GADD34, which is induced by cellular stress and has a central role in the unfolded protein response), and *ATF5* (encodes activating transcription factor 5, which regulates genes involved in mitochondrial protein homeostasis to protect against the mitochondrial unfolded protein response) were upregulated by poly(I:C) stimulation in control astrocytes, the same degree of upregulation was not present in VWMD cells (Figure 2A). Meanwhile, *DDIT3* (encodes CHOP, a pro‐apoptotic transcription factor, which is induced by the integrated stress response) appeared to increase with poly(I:C) treatment in VWMD cells and not control cells (Figure 2A). However, this effect was not observed for *ATF4* (activating transcription factor 4, also involved in the integrated stress response) expression (Figure 2A). This gene expression panel data suggests that VWMD astrocytes may have a diminished inflammatory response to double‐stranded RNA, which has been previously observed in *Eif2b5* ^R132H/R132H^ mice in response to bacterial lipopolysaccharide (LPS).^28^ The increase in *DDIT3* may be a preliminary indication of a pro‐apoptotic response to inflammation, which could trigger an initial demyelination event in VWMD patients.^29^ Further to this, gene responses of astrocytes following a scratch wound event were quantified in VWMD astrocytes compared to control astrocytes. *IL6*, *ATF4*, and *ATF5* were upregulated in VWMD astrocytes following scratch wounds, compared to controls (Figure 2B). However, there were no differences in *PP1R15A, DDIT3*, or mitochondrial markers, including copy number marker, *MT‐RNR2*, mitochondrial biogenesis markers, *PGC1A* and *SIRT1*, and autophagy marker, *MAP1LC3B* (Figure 2B). VWMD astrocytes also exhibited decreased *GFAP* (encodes a glial fibrillary acidic protein, a marker of astrocyte activation) expression after wound recovery compared to controls (Figure 2B).

As VWMD astrocytes have shown differential susceptibility to chemically induced endoplasmic reticulum (ER) stress,^19^ the differential response of VWMD astrocytes to ER stress was investigated. Basal expression levels of *IL6* were diminished in VWMD astrocytes compared to controls and further decreased by ER stress (Figure 2C). An increase in *PPP1R15A* (GADD34) and *DDIT3* (CHOP) was observed in VWMD cells compared to controls with MG132 stress, while *ATF4* and *ATF5* expression was unchanged. Additionally, there was a small but significant decrease in *MT‐RNR2* in VWMD patient cells compared to controls in the presence of MG132 stress (Figure 2C). The mitochondrial biogenesis markers, *PGC1A*, *SIRT1*, and autophagy marker, *MAP1LC3B,* showed small but significant increases in VWMD astrocytes, compared to controls, under MG132 stress (Figure 2C). Meanwhile, VWMD astrocytes showed decreased *GFAP* mRNA expression under both the presence and absence of ER stress (Figure 2C).

The gene expression data are in agreement with differentially upregulated GADD34 and CHOP under ER stress, as observed in patient histopathology^30^ and VWMD astrocyte cell culture.^19^ Dysfunctional mitochondrial protein expression has been proposed recently in primary VWMD animal model astrocytes.^10^ Therefore it was hypothesized that the expression of mitochondrial biogenesis regulators may be modulated in VWMD in order to compensate for dysfunctional energy homeostasis, however evidence of this was not observed here by RT‐qPCR. The decreased *IL6* and *GFAP* expression in VWMD astrocytes under unstressed conditions and with MG132 treatment suggest that this VWMD phenotype is further exacerbated by stress. *MAP1LC3B* (LC3B), responsible for autophagosome biogenesis, is a commonly utilized autophagy marker. The data suggest there was a comparable increase in response to ER stress.^31^ Although *ATF4* is anticipated to be upregulated by alternative splicing during stress‐induced eIF2B segregation, cell culture models have typically demonstrated a lack of differential *ATF4* upregulation under basal and chemically induced stress models.^13^

### 3.2 Differential protein expression of VWMD astrocytes

Vanishing white matter disease and control astrocytes were compared by SWATH‐MS to identify major proteomics level changes. This analysis was also used to investigate the potential for two therapeutic strategies to reverse pathway changes in VWMD astrocytes. Comparative proteomic analysis by functional gene ontology and IPA was performed using a SWATH‐MS spectral library of 2920 proteins with FDR ≤ 1%. The top 80 modulated proteins in VWMD cells (Figure S3) primarily included extracellular matrix, adhesion, and cytoskeletal proteins, particularly the collagen family (*COL18A1*, *COL1A2*, *COL5A2*, *COLGALT*), fibronectin (*FN1*), keratin (*KRT19*, *KRT7*, *KRT8*) integrins (*ITGA11* and *ITGA3*), surface proteoglycans glycipans (*GPC1*, *GPC4*), syndecan (*SDC2*), intercellular adhesion molecule 1 (*ICAM1*), microtubule‐associated proteins (*MAP1A*, *MAP1B*, *MAPRE3*) and vinculin (*VCL*). Top modulated proteins also included insulin‐like growth factor‐binding proteins (*IGFBP3*, *IGFBP5*, *IGFBP7*), downregulated chromatin remodeling histone cluster 1 H1 (*H1‐4, H1‐5*), and cell cycle‐associated proteins cyclin A2 (*CCN1*), pyrophosphatase 1 (*PPA1*), protein phosphatase 1 regulatory subunit 14C (*PPP1R14C*) and serine/threonine‐protein phosphatase 2A 56 kDa regulatory subunit epsilon (*PPP2R5E*). The top upregulated proteins also included large and small ribosomal units (*RPL13A*, *RPS9*), protein folding associated disulfide‐isomerases A3 and A6 (*PDIA3* and *PDIA6*),^32^ and heat shock proteins B8 and heparan sulfate proteoglycan 2 (*HSPB8* and *HSPG2*). Expression of ferritin light chain (*FTL*) and cytochrome b reductase 1 (*CYBRD1*), iron regulating proteins were also found to be modulated, along with the downregulation of metallothionine proteins (*MT1M*, *MT1X*, *MT2A*).

**FIGURE 3:**
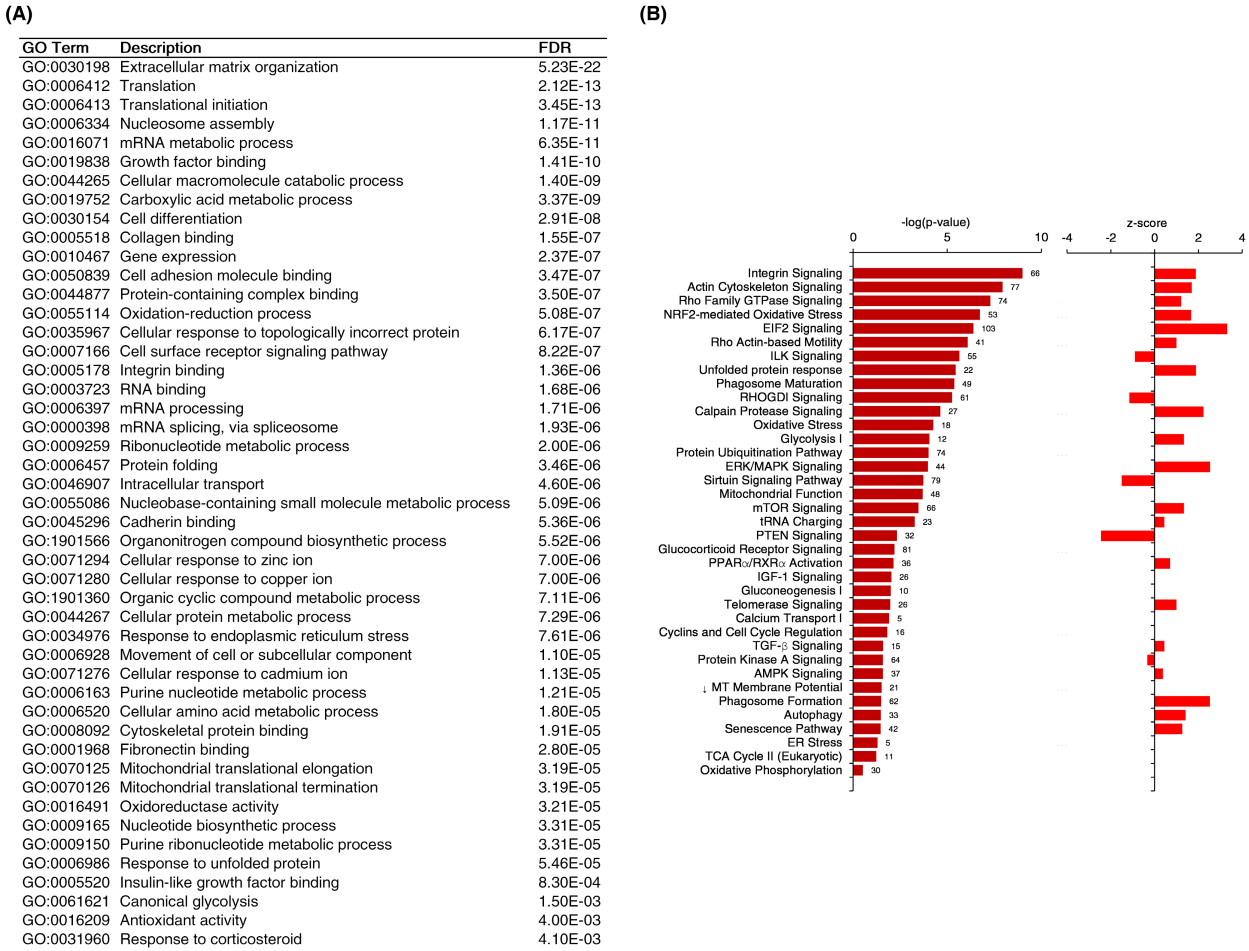
Gene ontology of vanishing white matter disease (VWMD) astrocyte differentially expressed proteins. (A) Canonical gene ontology terms, and (B) –log(p‐value) of IPA canonical pathways labeled with numbers of proteins in the pathways affected in VWMD *Z* activation scores (*Z*‐scores denote the number of standard deviations above or below the mean, *Z*‐score positive values are predicted upregulation, negative values predicted downregulation).

Gene ontology categories enriched by protein–protein interaction clusters in VWMD compared to control astrocytes included small molecule metabolism, extracellular matrix organization, cytoskeletal protein binding, ribosomal subunit, nuclear chromatin, response to unfolded protein, mitochondrial ribosome, metallothionine expression, and antioxidant activity and response to corticosteroids (Figure 3 and Figure S4). IPA canonical pathways identified included integrin signaling, EIF2 signaling, oxidative stress, gluconeogenesis, glycolysis, TCA cycle, OXPHOS, mitochondrial function, tRNA charging, unfolded protein response, autophagy, ER stress, and senescence. Importantly, downregulated mitochondrial membrane potential was also predicted by IPA, which correlated with the assay data in vitro (Figure 1).

Cytoskeletal proteins, such as actin, represent over 5% of total cellular protein,^33^ while astrocytes secrete extracellular matrix proteins, particularly collagen.^34^ As such, the continuous burden of structural protein turnover due to cellular stress may be affected by, and further exacerbate the VWMD cellular phenotype. Cytoskeletal proteins are also involved in vesicle formation and membrane remodeling, and thus potentially affect autophagy. VWMD astrocytes showed enlarged flattened morphology in high‐viscosity alginate medium and upregulated proteins involved in migration (Figure S1).

Low expression of histone H1 family proteins may reflect a loss of chromatin compaction, which can impede neural differentiation.^35^ Observed downregulation of tryptophanyl‐tRNA synthetase 1 (*WARS1*)^36^ and N‐myc downstream regulated 1 (*NDRG1*) has been observed in cancerous phenotypes,^37^ as well as upregulated pro‐proliferative membrane‐associated progesterone receptor component 1 (*PGRMC1*).^38^ Upregulated proliferative pathways may be correlated to the lack of astrocyte differentiation and increases in immature glial progenitors, as observed in VWMD post‐mortem tissue. This is complemented by the increased levels of cluster of differentiation 44 (*CD44*), potentially indicating neural progenitor immaturity,^39^ along with upregulated nestin (*NES*), while loss of dihydropyrimidinase‐related protein 2 (*DPYSL2*) has also been associated with poor neural stem cell differentiation.^40^ Upregulated *CD44* expression has also been previously identified in VWMD post‐mortem tissue, as well as iPSC‐derived VWMD astrocytes.^9^

Metallothionines bind to physiological and exogenous metal ions and, as antioxidants, mitigate oxidative stress.^41^ The downregulation of metallothionines (*MT1M*, *MT1X*, *MT2A*), as well as catalase (*CAT*) in VWMD astrocytes, may contribute to the observed elevated level of reactive oxygen species. Additionally, the IPA‐predicted decrease in mitochondrial membrane potential agrees with the decreased TMRE staining of VWMD astrocytes experimentally obtained here and as observed in Eif2b^R132H^/^R132H^ mice.^10^ Downregulated *MT2A* has been previously identified in iPSC‐derived astrocytes.^9^

Insulin growth factor 1 (IGF‐1) secretion by glial cells has been linked to oligodendrocyte precursor cell maturation.^5^ IGF‐1 is also neuroprotective, partly by mitigating oxidative stress in astrocytes and facilitating the reduction of hydrogen peroxide‐induced stress in neuronal cocultures.^42^ Modulation of IGF‐1 binding protein levels may disrupt this process given the multiple roles of IGF binding proteins to both inhibit the IGF receptor interaction but also prolonging bioavailability in circulation.^43^

### 3.3 Edaravone treatment of VWMD astrocytes

**FIGURE 4:**
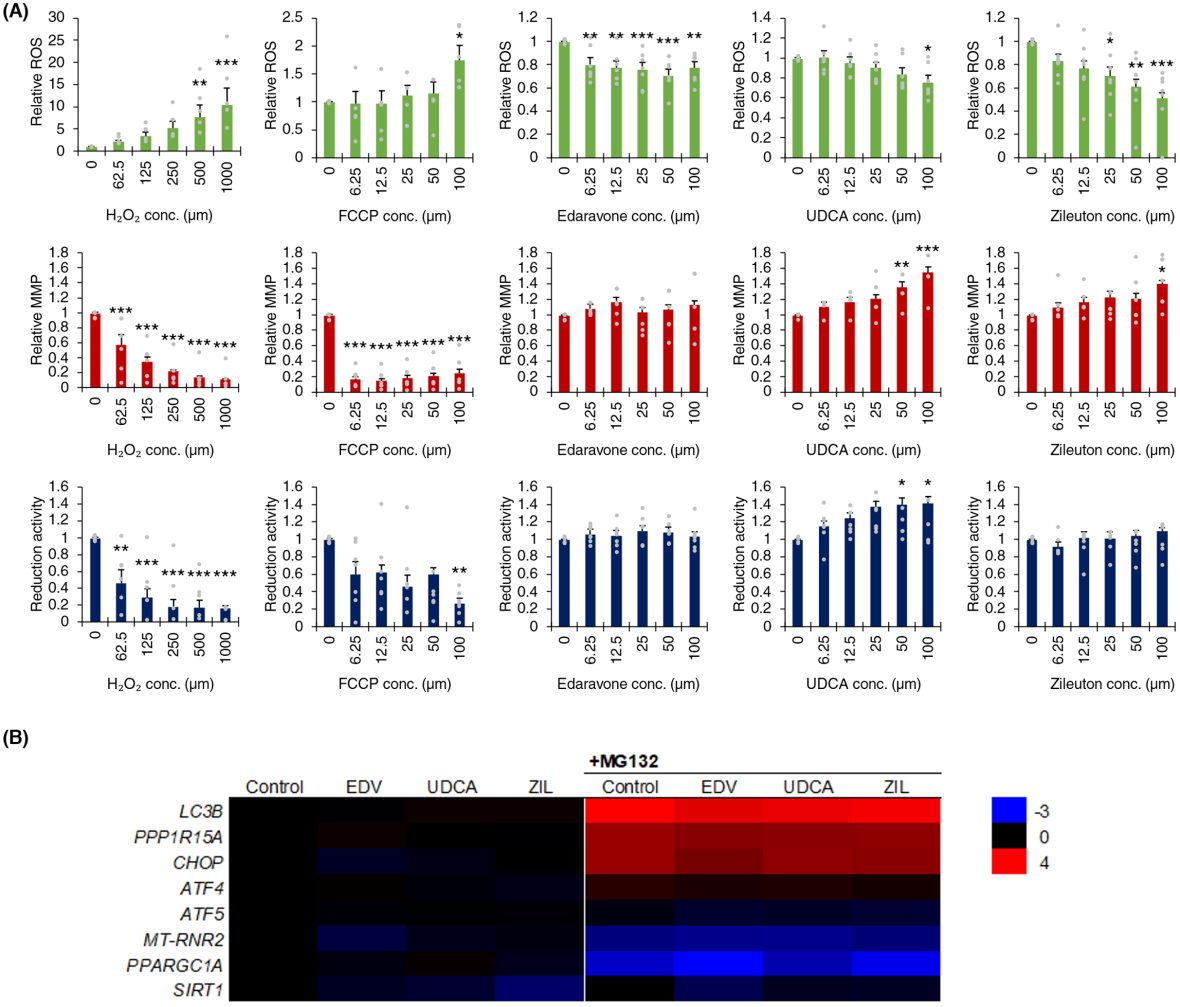
Effect of edaravone, UDCA, and zileuton on reactive oxygen species, mitochondrial membrane potential, and reduction activity in vanishing white matter disease (VWMD) astrocytes (*EIF2B2*). (A) Dose‐response assay demonstrated intrinsic effects of edaravone, UDCA, and zileuton in VWMD astrocytes (*n* = 6). (B) Gene expression of integrated stress response genes, mitochondrial biogenesis, mitochondrial content, and autophagy markers of candidate compounds at lowest concentrations determined to have an antioxidant effect (edaravone, 10 μM, zileuton 25 μM) or ΔΨm (UDCA, 50 μM) in astrocytes under unstressed and ER stressed conditions (0.5 μM MG132; *n* = 4). Data presented as mean ± SEM, * *p* < 0.05, ** *p* < 0.01, *** *p* < 0.001 by one‐way ANOVA with Holm‐Sidak post hoc multiple comparisons test.

We previously identified a number of anti‐inflammatory compounds that may ameliorate cytotoxic ER stress in VWMD astrocytes.^19^ However, the potential adverse effects of both glucocorticoids and non‐steroidal anti‐inflammatory drugs (NSAIDs) preclude their long‐term usage to treat CNS diseases; long‐term glucocorticoid usage can be psychoactive,^44^ while NSAID usage significantly increases the risk of ischemic stroke^45^ and cardiovascular complications.^46^ Edaravone, ursodiol (UDCA), and zileuton were screened in VWMD astrocytes for their effect on oxidative stress, ΔΨm, reduction activity, and gene expression of ISR, mitochondrial biogenesis, and autophagy markers. Edaravone significantly decreased ROS generation at concentrations ≥6.25 μM, while zileuton showed similar activity at ≥25 μM (Figure 4A). Hydrogen peroxide and FCCP, which uncouples mitochondrial oxidative phosphorylation, were used as positive controls for oxidative stress, mitochondrial membrane potential and reduction activity assays.

**FIGURE 5:**
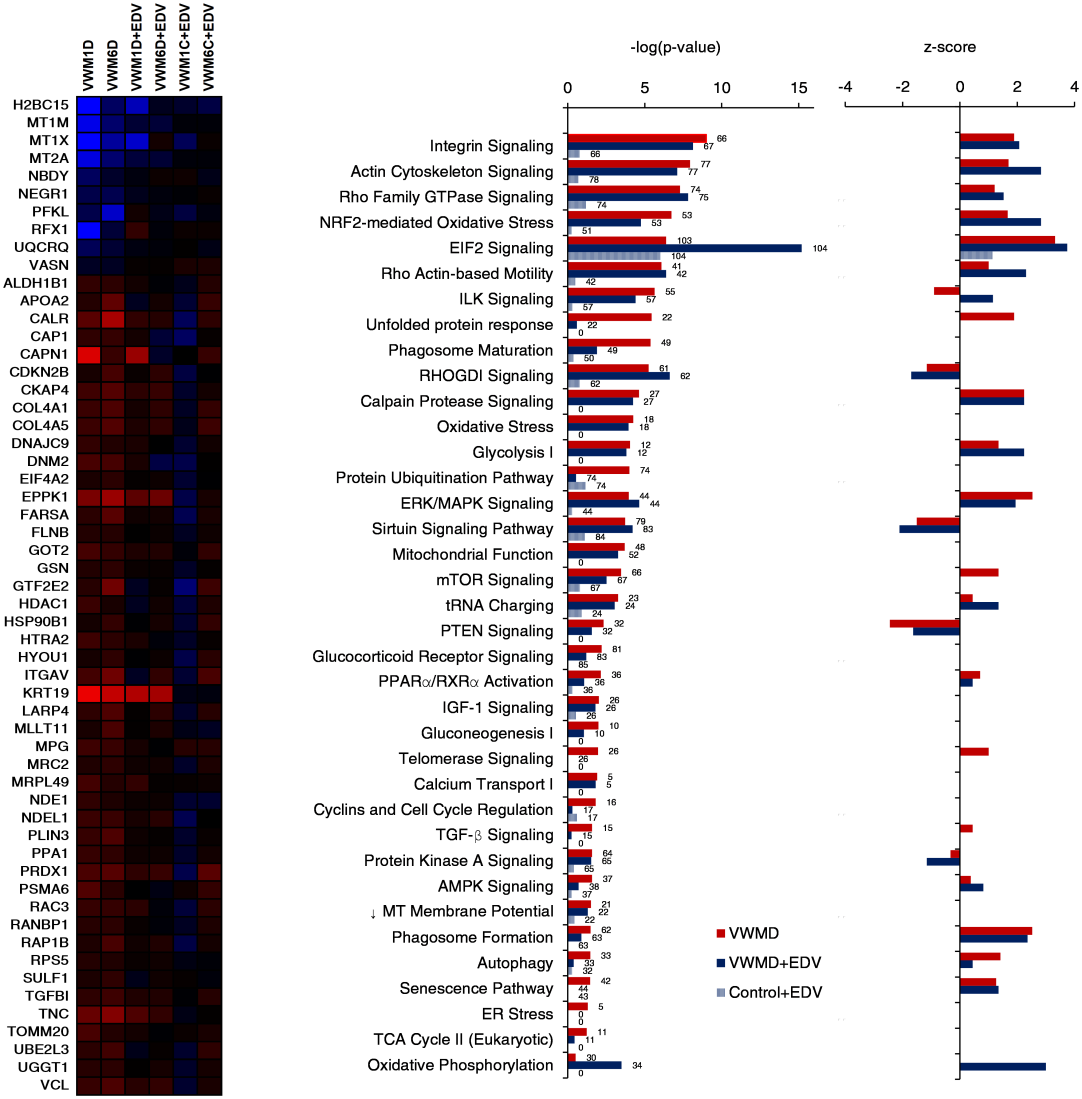
Heatmap demonstrating vanishing white matter disease (VWMD) astrocyte protein expression altered by edaravone (EDV) compared to control astrocyte +EDV and canonical pathway gene enrichment and predicted directional activation changes. The –log(*p*‐value) of IPA canonical pathways labeled with numbers of proteins in the pathways that were affected and *Z*‐scores in VWMD (red) compared to VWMD + EDV (blue). Control astrocytes + EDV are shown for reference (*n* = 3).

Mitochondrial membrane potential (ΔΨm) was upregulated by UDCA ≥50 μM, as well as causing an increase in reduction activity. Under ER stress conditions, edaravone significantly reduced upregulated expression of pro‐apoptotic *DDIT3* but had no significant effect on autophagy marker *MAP1LC3B* (Figure 4B). There was no effect of edaravone on the protein synthesis rate of VWMD or control astrocytes under basal or stressed conditions (Figure S2). Given the ROS‐scavenging properties of edaravone and its capacity to ameliorate ER stress,^47^ the effect of edaravone on VWMD astrocytes was investigated by proteomic analysis (Figure 5). The lack of effect of edaravone on mitochondrial membrane potential or reduction activity suggests that its mode of action as an antioxidant does not significantly alter ATP generation activity. Gene ontology clustering identified changes induced by edaravone in VWMD astrocytes that included pathways involved in cellular and mitochondrial translation, cell structure and motility, nuclear chromatin, extracellular matrix organization, small molecule metabolic processes, response to unfolded protein, mitochondrial electron transport, and metallothionines (Figure S5).

Proteins that were upregulated by edaravone treatment included antioxidant metallothionines (*MT1M*, *MT1X*, and *MT2A*),^41^ complex III cytochrome b‐c1 subunit (*UQRCQ*), and part of the electron transport chain and phosphofructokinase (liver; *PFKL*; Figure 5). Proteins elevated in VWMD astrocytes that were downregulated by edaravone treatment included extracellular matrix and cytoskeletal associated proteins collagen IV alpha chain (*COL4A1*), (*COL4A5*), integrin alpha‐V (*ITGAV*), keratin (*KRT19*), vinculin (*VCL*), nuclear distribution proteins (*NDE1*, *NDEL1*; Figure 5).

Edaravone treatment also downregulated: mitochondrial aldehyde dehydrogenase 1 family member B1 (*ALDH1B1*), which is upregulated in response to lipid oxidation;^48^ perilipin‐3 (*PLIN3*), known to be upregulated in intracellular droplets during aging;^49^ peroxiredoxin‐1 (*PRDX1*), an antioxidant protein and marker of resistance to oxidative stress;^50^ as well as large and small ribosomal subunit proteins (*RPL9*) and (*RPS5*), mitochondrial ribosomal protein (*MRPL49*), mitochondrial import receptor subunit (*TOMM20*) homolog (Figure 5). Protein folding chaperone calreticulin (*CALR*), cytoskeleton proteolysis catalyst calpain‐1 (*CAPN1*),^51^ aspartate aminotransferase (*GOT2*), and proteotoxic stress‐related 20S core proteasomal complex subunits (*PSMA2*, *PSMB2*, *PSMA5*, *PSMA6*), and ubiquilin‐2 (*UBQLN2*),^52^ were also decreased by edaravone.

Ingenuity Pathway Analysis of VWMD astrocytes indicated that canonical pathways, including the unfolded protein response (UPR), phagosome regulation, ubiquitination pathway, autophagy, ER stress, telomerase signaling, cell cycle regulation, TGF‐β signaling, senescence, TCA cycle II and oxidative phosphorylation were modulated by edaravone. Overall, edaravone‐treated VWMD astrocytes were less impacted by the VWMD phenotype, compared to untreated astrocytes.

### 3.4 Mitochondrial transfer in VWMD astrocytes

**FIGURE 6:**
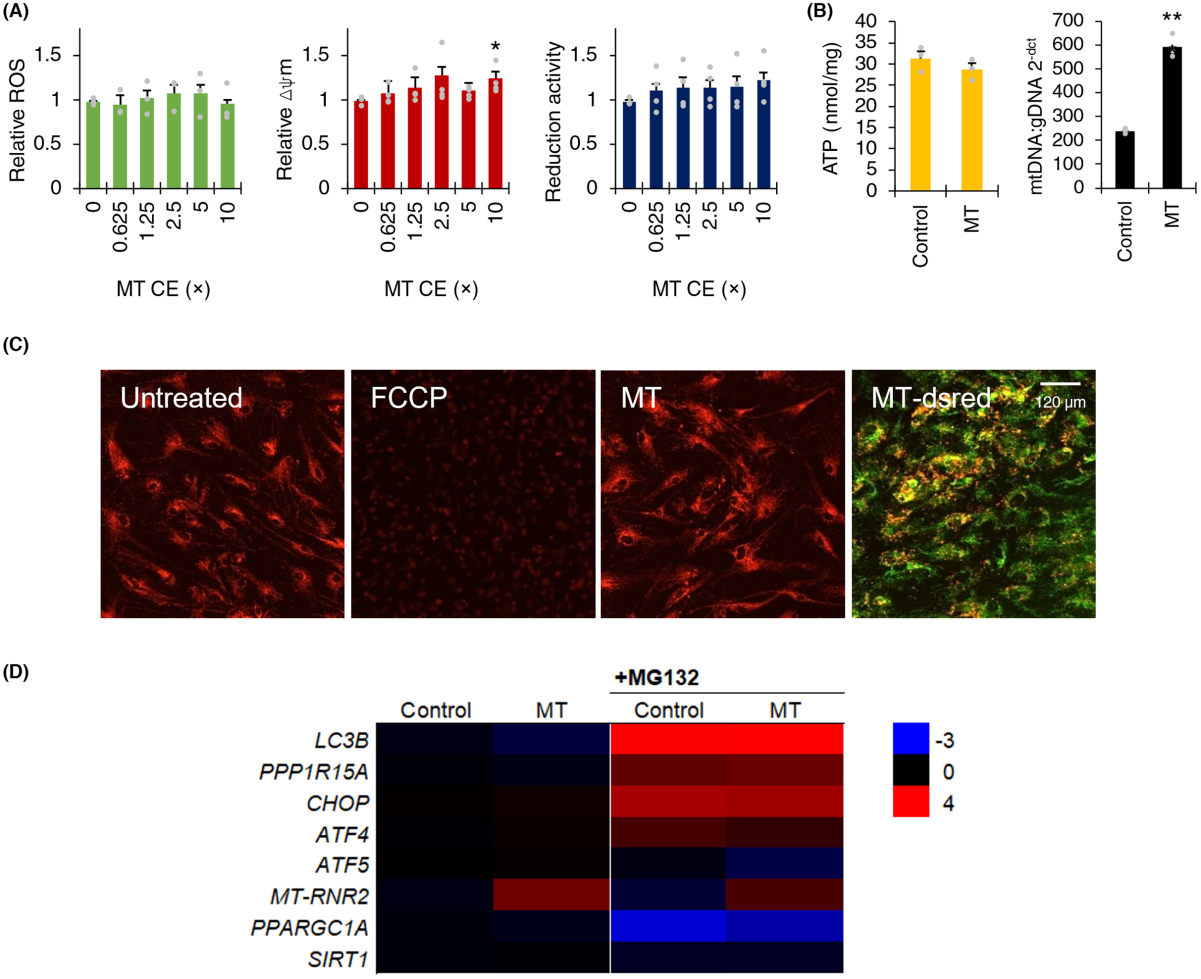
Mitochondrial transfer in vanishing white matter disease (VWMD) astrocytes. (A) Oxidative stress (ROS generation), mitochondrial membrane potential (∆ψm), and reduction activity by mitochondrial transfer (MT) with 0.625–10 cell equivalents (CE). (B) Intracellular ATP and relative mitochondrial DNA (mtDNA) following MT (*MT‐RNR2* relative to *GAPDH*). (C) Representative images of mitochondrial transfer in VWMD astrocytes stained with TMRE, and overlay of MT with mitochondria‐localized DsRed with Rhodamine 123. (D) Effect of MT on integrated stress response markers, mitochondrial copy level, mitochondrial biogenesis, and autophagy markers. Data presented as mean ± SEM (*n* = 3), * *p* < 0.05, ** *p* < 0.01, *** *p* < 0.001 by one‐way ANOVA with Holm‐Sidak post hoc multiple comparisons test.

Given the prevalence of mitochondrial astrocytic dysfunction in VWMD, we investigated whether mitochondrial transfer could be used to ameliorate the disease phenotype. Live mitochondrial extraction and buffer conditions were optimized as described in the Section 2 (Methods) and Supplementary data (Figure S7). Migration of mitochondria was observed (Figure S7) and appeared cytosolic in localization (Figure 6C, Figure S8). Mitochondrial transfer led to a significant increase in mitochondrial membrane potential, mitochondrial DNA, and *MT‐RNR2* gene expression, with no significant changes in oxidative stress, resazurin reduction activity, or intracellular ATP levels (Figure 6A‐C). Mitochondrial transfer did not significantly affect the unfolded protein response markers, *PPP1R15A*, *ATF4*, *ATF5*, *DDIT3*, or autophagy marker, *MAP1LC3B* (Figure 6D). Under ER stress, elevated *ATF5* and *DDIT3* were decreased (Figure 6D). No change in protein synthesis rate was observed under basal or stressed conditions by mitochondrial transfer (Figure S2).

The increase in mitochondrial: genomic DNA by qPCR, upregulation of *MT‐RNR2,* detected by RT‐qPCR following mitochondrial transfer, and localization of fluorescent‐tagged exogenous mitochondria together indicated that mitochondrial uptake occurred within an overnight period, and remained cytosolically localized for at least 48 h (Figure S8). Although mitochondrial transfer involves the uptake of mitochondria and their associated proteins, interestingly no induction of ER stress or autophagy marker expression changes were evident from the uptake of mitochondrial extract, which suggests that the intake of transferred organelle did not include damaged proteins. The absence of change in oxidative stress, reduction activity, or intracellular activity indicates that intracellular ATP homeostasis was retained.

As mitochondrial transfer appears to be feasible without exogenous agents,^53^ we considered the potential for mitochondrial transfer as a gene delivery vehicle. Mitochondria electroporated with a DNA plasmid^22^ were incubated with VWMD astrocytes, however, the efficiency of reporter expression was found to be barely detectable (Figure S9). Based on these data, this approach was not pursued further as a mode of gene delivery.

**FIGURE 7:**
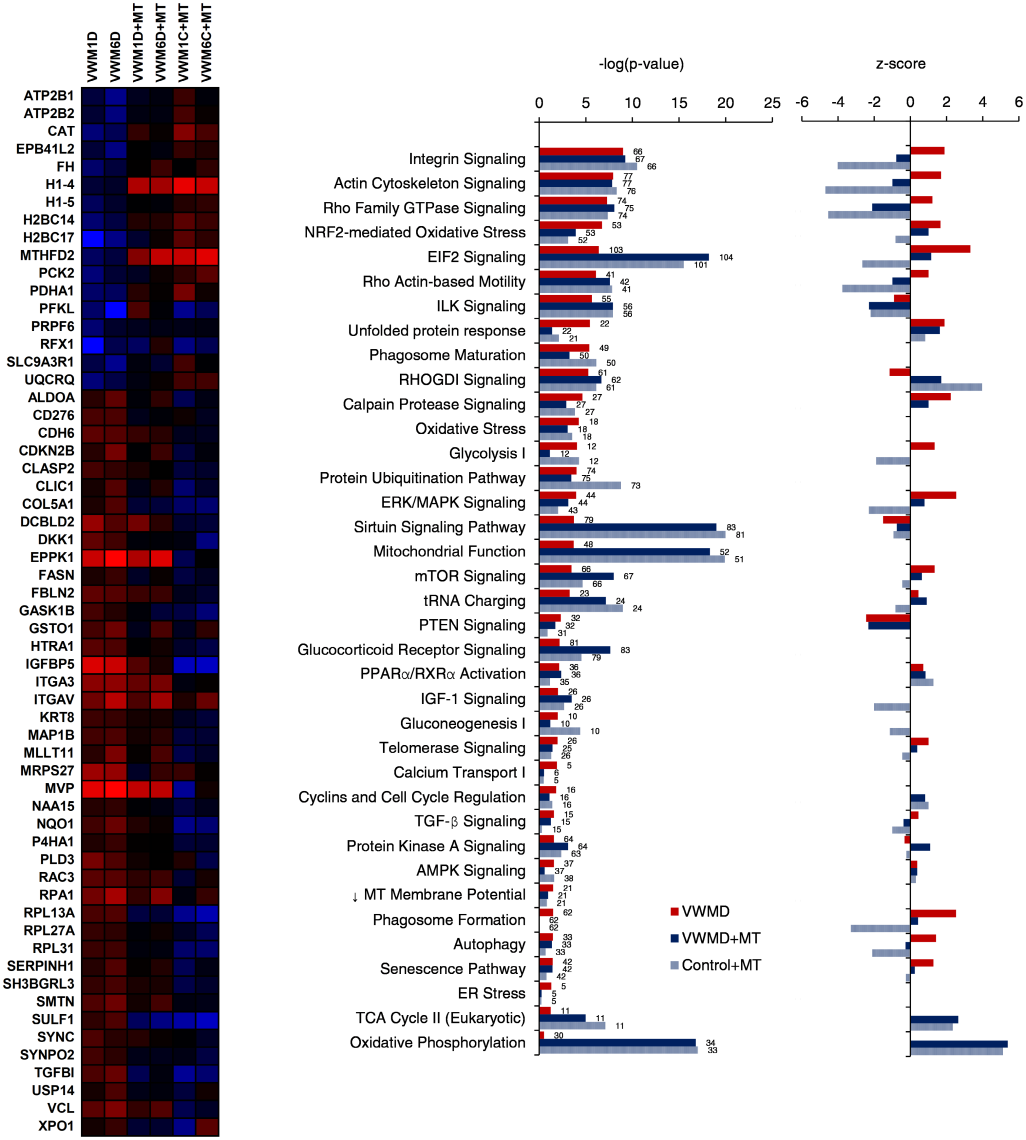
Vanishing white matter disease (VWMD) protein expression altered by mitochondrial transfer, canonical pathway gene enrichment, and directional activation changes. Top proteins differentially affected by mitochondrial transfer, and alternations in IPA (*n* = 3).

The effect of mitochondrial transfer on VWMD astrocytes was further investigated by proteomic analysis (Figure 7). Gene ontology‐enriched clusters included oxidative phosphorylation, mitochondrial ribosome, chromatin organization, ribosomal subunit, ribonucleoprotein complex, and endoplasmic reticulum stress (Figure S6). Several cytoskeletal and structural proteins upregulated in VWMD astrocytes were found to be downregulated by mitochondrial transfer (Figure 7), including collagen alpha‐1(V) chain (*COL5A1*), epiplakin (*EPPK1*), smoothelin (*SMTN*), extracellular sulfatase Sulf‐1 (*SULF1*), synaptopodin‐2 isoform 4, which induces actin bundles (*SYNPO2*), vinculin, actin filament (F‐actin)‐binding protein, involved in cell‐matrix adhesion and cell–cell adhesion (*VCL*), and serine protease HtrA Serine Peptidase 1 (*HTRA1*), which targets extracellular matrix proteins, such as fibronectin.^54^

Downregulated VWMD proteins that were upregulated by mitochondrial transfer included cytochrome B‐C1 complex subunit 8 (*UQCRQ*) a complex III subunit, plasma membrane calcium‐transporting ATPases (*ATP2B1*, *ATP2B2*), which may be in response to increased ATP levels in order to retain homeostasis, hydrogen peroxide antioxidant catalase (*CAT*)^55^ (Figure 7). Upregulated proteins also included metabolic proteins, including amino acid processing mitochondrial methylenetetrahydrofolate (*MTHFD2*), glycolysis ATP‐dependent 6‐phosphofructokinase (*PFKL*), which catalyzes the first stage of glycolysis, mitochondrial pyruvate dehydrogenase E1 subunit alpha (*PDHA1*; catalyzes pyruvate to acetyl‐CoA, linking glycolysis and TCA cycle), and mitochondrial fumarate hydratase (*FH*), which catalyzes fumarate to L‐malate in the TCA cycle. Elevated VWMD expression of aldolase fructose‐bisphosphate A (*ALDOA*), which catalyzes fructose‐1,6‐bisphosphate to glyceraldehyde 3‐phosphate (*G3P*), and dihydroxyacetone phosphate (*DHAP*) were decreased by mitochondrial transfer (Figure 7).

Chromatin regulating histone cluster H1 (*H1‐4*, *H1‐5*) and H2B family proteins (*H2BC14*, *H2BC17*) that were lower in abundance in VWMD astrocytes, were upregulated by mitochondrial transfer. Mitochondrial transfer reduced the abundance of insulin‐like growth factor‐binding protein 5 (*IGFBP5*), transforming growth factor‐beta‐induced protein (*TGFBI*), which upregulates proliferation and inflammation^56^ (Figure 7). Elevated immune costimulatory protein CD276 (*CD276*) correlated with proliferation in VWMD was decreased by mitochondrial transfer.^57^ Mitochondrial transfer also decreased elevated levels of dickkopf‐related protein 1 (*DKK1*) a Wnt signaling antagonist that at high expression is known to inhibit neurogenesis^58^ (Figure 7).

Nucleic acid and protein processing proteins upregulated in VWMD that were reduced in expression by mitochondrial transfer included major vault protein (*MVP*), which facilitates mRNA and protein packaging/transport,^59^ mitochondrial ribosomal protein S27 (*MRPS27*), large subunit ribosomal proteins l13ae, l27ae, l31e (*RPL13A*, *RPL27A*, *RPL31*) and replication protein A DNA‐binding subunit (*RPA1*). Additionally, mitochondrial transfer downregulated protein regulation proteins Golgi‐associated kinase 1 B (*GASK1B*), phospholipase D3 (*PLD3*) a neuronal lysosomal protein that aggregates in plaques,^60^ and exportin‐1 which mediates the nuclear export of cellular proteins (*XPO1*)^61^ (Figure 7).

Ingenuity Pathway Analysis indicated that mitochondrial transfer increased differential pathways of EIF2 signaling, sirtuin signaling, tRNA signaling, TCA cycle, and OXPHOS proteins relative to VWMD and control astrocytes, mitigated VWMD changes in the UPR, glycolysis, calcium transport, phagosome formation, and ER stress pathways, while also inhibiting VWMD aberrantly activated integrin, actin, GTPase signaling, and autophagy pathways (Figure 7).

### 3.5 Mitochondrial transfer may enhance the maturation of VWMD astrocytes

**FIGURE 8:**
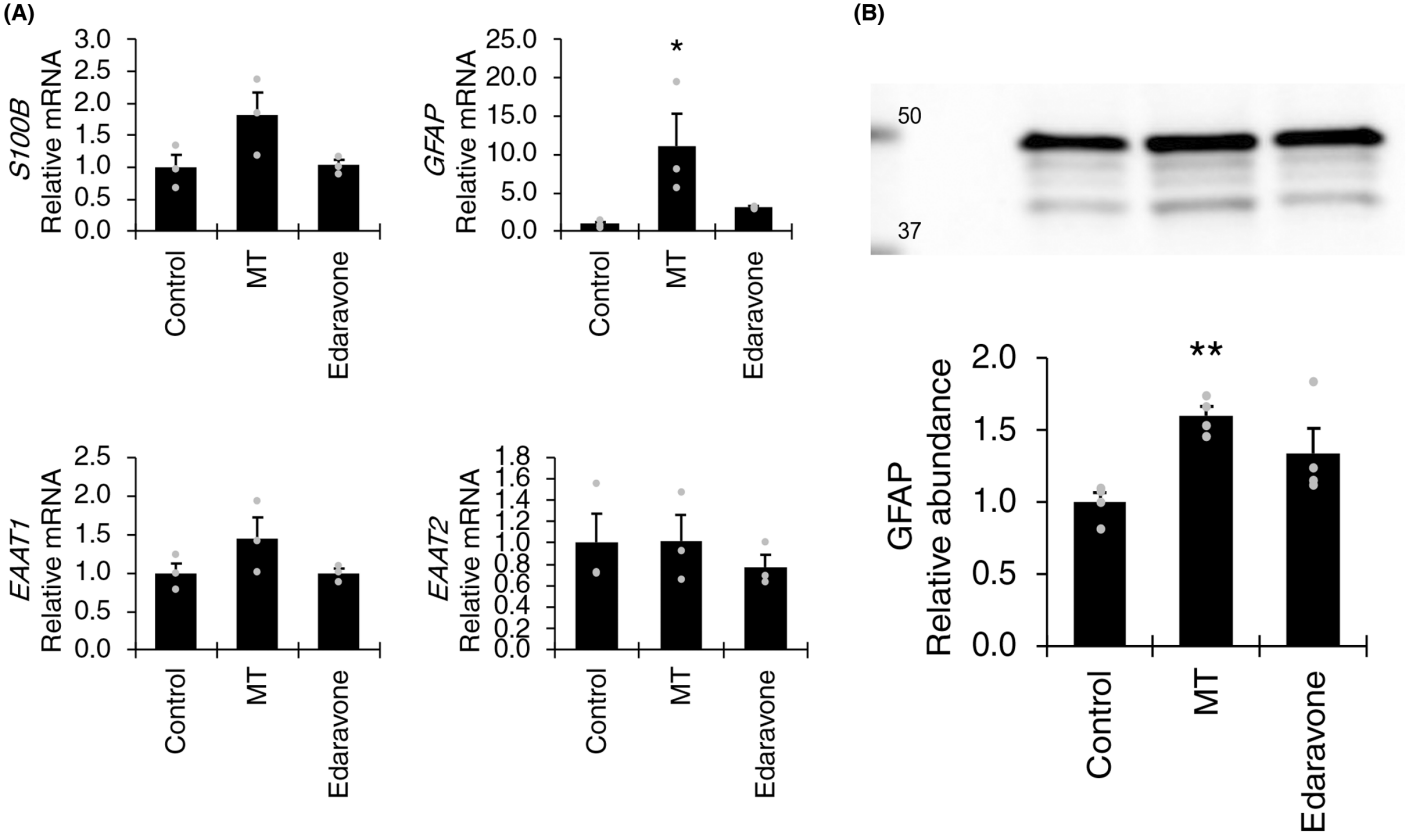
Effect of mitochondrial transfer and edaravone on differentiation of vanishing white matter disease (VWMD) astrocytes. (A) RT‐qPCR of *S100B*, *GFAP*, *EAAT1*, and *EAAT2* mRNA expression changes (*n* = 3) and (B) GFAP western blot (*n* = 4). Data presented as mean ± SEM * *p* < 0.05, ** *p* < 0.01, by one‐way ANOVA with Holm‐Sidak post hoc multiple comparisons test.

As the development of VWMD has been identified to be critically affected by the maturation of astrocytes^9^ that support the survival and differentiation of white matter myelin‐generating oligodendrocytes, we assessed the effect of mitochondrial transfer and edaravone on the maturation of VWMD astrocytes (Figure 8). Although there was no significant difference in GFAP mRNA and protein expression by coincubation with edaravone, a significant increase in GFAP mRNA and protein expression was observed by mitochondrial transfer. These data suggest that astrocyte maturity may be enhanced by mitochondrial transfer, however further studies are required to confirm whether this effect on the neural precursor to astrocyte differentiation could occur at various stages of the disease in vivo.

## 4 CONCLUSIONS

Overall, data from this study agree with the canonical characterization of edaravone as a potent free radical scavenger and highlights its potentially protective value in the VWMD phenotype of oxidative stress. Thus, in VWMD, edaravone has the potential for alleviating abnormal unfolded protein response, protein ubiquitination and autophagy pathways, and increasing oxidative phosphorylation signaling. The antioxidant effect of edaravone has been reported to block ubiquitin accumulation and proteasomal inhibition‐associated cell death.^62^ As a repurposable drug, edaravone could be considered as an anti‐proteotoxic, cytoprotective drug against cell death observed in chronic disease progression of VWMD,^63^ not dissimilar to its approved usage in ALS, and its recent approval for oral usage may support its implementation by way of decreasing risk of infection, patient discomfort and cost.^64^

Mitochondrial transfer was also found to increase oxidative phosphorylation signaling, while downregulating aberrant glycolysis, unfolded protein response, cell structure, and motility signaling pathways. Interestingly, an increase in reduction activity and ATP generation, as previously observed in the literature,^53^, ^65^ was not observed, potentially due to comparatively lower mass of loaded mitochondria, or rapid homeostasis of ATP. The premise of mitochondrial transfer for VWMD also includes its possibility of enhancing astrocyte maturation. However, there are a number of challenges to retaining mitochondrial viability demonstrated in cell culture; mitochondrial optimization experiments suggest that isolated mitochondria are not necessarily bioenergetically active after several hours at 37°C, and highlight the sensitivity of mitochondria to divalent ions once extracted (Figure S7). Mitochondrial transfer is an emerging and promising organelle replacement therapy,^65^ as mitochondria are able to enter the CNS through intravenous^66^ and intranasal^67^ delivery methods. Since electroporation of mitochondria has been demonstrated for the correction of mitochondrial disorders,^22^ it was also plausible that mitochondria could be utilized as an exogenous gene delivery vehicle, potentially for replacing levels of wild‐type EIF2B1–5 subunits, however, preliminary experiments suggest further optimization is required to enable this. Optimization data agree that storage at 4°C can preserve the integrity of mitochondria for at least an overnight period (Figure S7), which is in agreement with the preservation of mitochondria in live tissue and isolated cells at low temperatures.^68^, ^69^ Furthermore, it has been demonstrated that mitochondria can be cryopreserved to retain outer membrane structure,^70^ enabling their usage as an inventoriable therapeutic.

Thus, edaravone and mitochondrial transfer should be further explored as potential therapeutics for VWMD and other diseases in which astrocyte oxidative stress, mitochondrial and proteostasis dysfunction are relevant.

## AUTHOR CONTRIBUTIONS

NN and LO designed the project. All authors edited the manuscript. NN, MN, AT, SM, and JC performed experiments and analyzed data. LC, TZ, and YW performed proteomics experiments and analyzed data. NN and LO analyzed data and prepared the manuscript.

## FUNDING INFORMATION

LO was supported by a National Health and Medical Research Council (NHMRC) of Australia Boosting Dementia Research Leadership Fellowship awarded (APP1135720). Gene expression assay reagents were supported by an IHMRI Young Investigator Grant (NN) and MAWA Research Grant (NN).

## CONFLICT OF INTEREST STATEMENT

The authors declare they have no conflicts of interest.

## Supporting information


Figures S1‐S10

## Data Availability

The datasets used and analyzed during the current study are available from the corresponding author upon reasonable request.
