# Supplementary material for: Edaravone and mitochondrial transfer as potential therapeutics for vanishing white matter disease astrocyte dysfunction
Source: CNS Neurosci Ther. 2023 Mar 27;29(9):2481–97. doi: 10.1111/cns.14190 (PMC10401142; doi:10.1111/cns.14190)
Supplement: Supplementary file 1 — Figures S1‐S10 [file CNS-29-2481-s001.pdf]

Edaravone and mitochondrial transfer as potential therapeutics for vanishing white matter disease astrocyte dysfunction

Supplementary Data

Neville S. Ng [a,b] *, Michelle Newbery [a,b], Aude Touffu [a,b], Simon Maksour [a,b], Johnson Chung [c], Luke Carroll [d], Thiri Zaw [d], Yunqi Wu [d], Lezanne Ooi [a,b] *

[a.] Illawarra Health and Medical Research Institute, Northfields Avenue, Wollongong, NSW 2522, AU.

[b.] School of Chemistry and Molecular Bioscience and Molecular Horizons, University of Wollongong, Northfields Avenue, Wollongong, NSW 2522, AU.

[c.] Intelligent Polymer Research Institute, ARC Centre of Excellence for Electromaterials Science, AIIM Facility, Innovation Campus, University of Wollongong, NSW, Australia

[d.] Australian Proteome Analysis Facility (APAF), Macquarie University, Research Park Dr, Sydney 2109 AU.

*Corresponding author: Dr Lezanne Ooi, University of Wollongong, Northfields Avenue, Wollongong, NSW 2522, Australia. Tel: +61242215865; Email: lezanne@uow.edu.au.

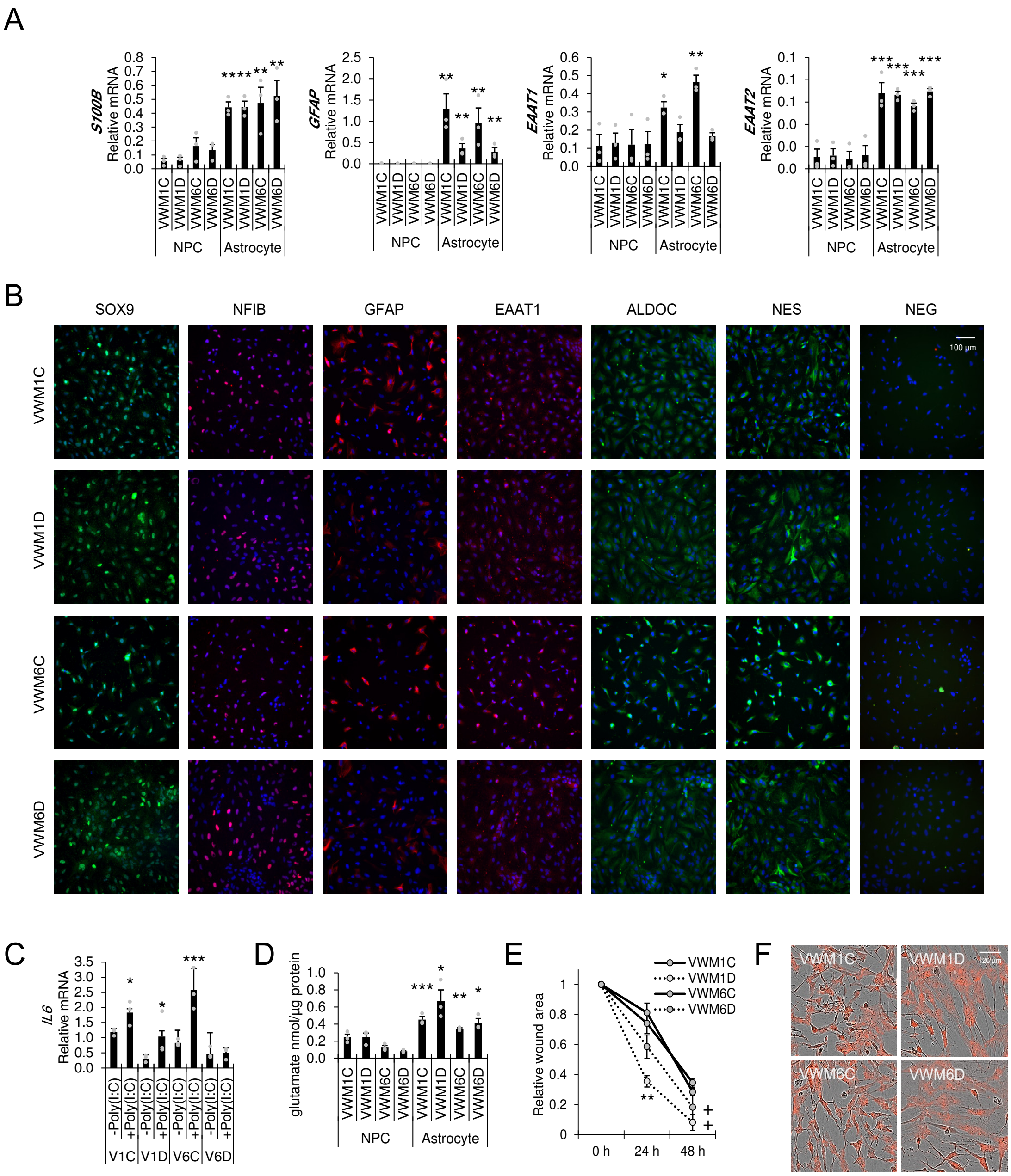

Figure S1. Gene expression, immunofluorescence, immunoreactivity, intracellular glutamate and wound-healing assay characterisation of VWMD and control astrocytes (day 12-15). Data presented as mean ± SEM (n = 3).

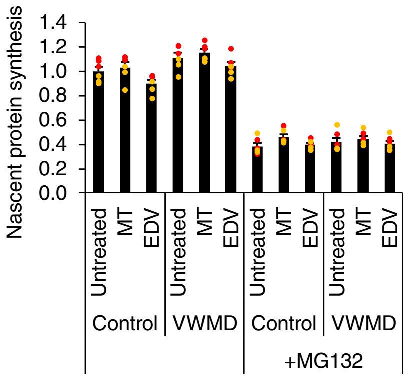

Figure S2. HPG protein synthesis assay with control and VWMD disease lines treated with mitochondrial transfer (MT), or edaravone. Data presented as mean ± SEM (n = 3) (red = *EIF2B5* disease and control and orange = *EIF2B2* disease and control line) (One-way ANOVA with Holm-Sidak post-hoc multiple comparisons test).

Gene name	log2(FC)	Adjusted p-value
AKAP12	-0.50	4.38E-02
APOL2	-0.44	4.27E-02
C11orf54	-0.71	4.83E-02
CCDC80	-0.92	4.03E-03
CCN1	-0.88	4.26E-02
COL1A2	-0.93	4.26E-02
COL5A2	-0.90	3.98E-02
CPSF6	-0.34	4.16E-02
CRIP2	-0.73	7.97E-03
CYBRD1	-0.84	3.89E-02
DNASE2	-0.77	4.26E-02
DPYSL2	-0.33	1.21E-02
ENAH	-0.80	4.27E-02
GFUS	-0.56	4.16E-02
GPC1	-0.88	3.26E-02
GPC4	-0.76	4.16E-02
H1-4	-0.32	2.82E-02
H1-5	-0.48	4.36E-02
ICAM1	-0.93	4.03E-03
IGFBP3	-1.43	3.87E-03
IGFBP7	-0.46	4.85E-02
ITGA11	-0.61	3.96E-02
KRT7	-0.53	2.27E-02
MT1M	-1.67	2.02E-02
MT1X	-2.81	4.50E-02
MT2A	-1.62	2.05E-02
NDRG1	-0.69	4.27E-02
PALM	-0.51	2.08E-02
PLS3	-0.82	4.16E-02
PPP1R14C	-0.78	3.20E-02
PTGIS	-1.11	2.82E-02
RAB18	-0.58	3.52E-02
RSRC1	-0.70	4.16E-02
SDC2	-0.50	4.16E-02
SLC3A2	-0.74	4.16E-02
SLC9A3R1	-0.75	4.16E-02
SLX9	-0.57	4.16E-02
SON	-0.55	4.78E-02
TCEA1	-0.76	4.83E-02
TCEA2	-0.76	4.16E-02
TYMP	-0.88	4.16E-02
VASN	-0.34	4.27E-02
WARS1	-0.67	4.27E-02

Gene name	log2(FC)	Adjusted p-value
AMIGO2	0.86	4.03E-03
CCDC50	0.63	4.16E-02
CD44	0.64	4.27E-02
CDH6	0.63	6.28E-03
COL18A1	1.02	3.25E-02
COLGALT1	0.67	4.26E-02
COX5A	0.34	3.52E-02
DCBLD2	0.86	4.26E-02
EPPK1	1.60	2.95E-02
FBLN2	0.63	4.16E-02
FN1	0.96	2.82E-02
FTL	1.19	4.27E-02
GOT2	0.73	4.38E-02
HSPB8	0.66	4.27E-02
HSPG2	0.51	4.16E-02
IGFBP5	1.52	3.87E-03
ITGA3	0.99	3.18E-02
ITPA	0.73	1.21E-02
KRT19	2.53	2.82E-02
KRT8	0.41	4.27E-02
MAP1A	0.35	2.29E-02
MAP1B	0.50	3.11E-02
MAPRE3	0.68	2.82E-02
MVP	2.12	3.03E-02
P4HA1	0.30	4.27E-02
PDIA3	0.32	3.87E-03
PDIA6	0.33	3.03E-02
PGRMC1	0.44	3.11E-02
PPA1	0.58	4.26E-02
PPP2R5E	0.36	4.16E-02
PRAF2	0.37	4.94E-02
RCN1	1.12	4.26E-02
RPL13A	0.53	4.16E-02
RPS9	0.57	3.20E-02
SMTN	0.67	4.27E-02
VCL	0.74	4.16E-02
VCPIP1	0.67	6.28E-03

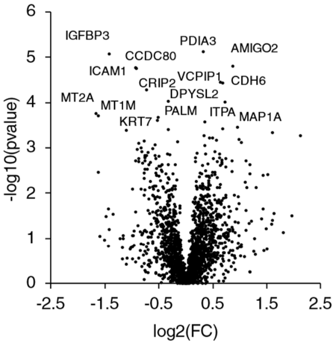

Figure S3. Top 80 differentially expressed VWMD astrocyte proteins.

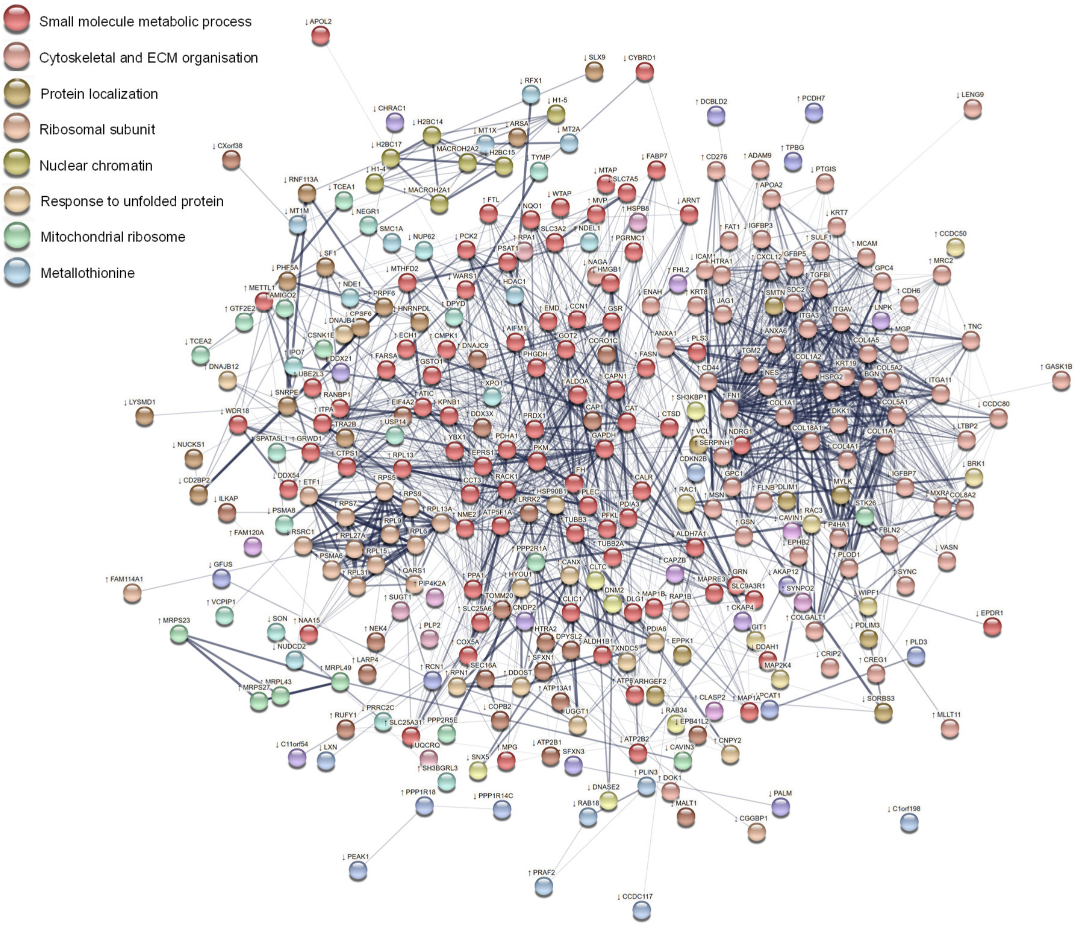

Figure S4. Differentially expressed VWMD astrocyte protein-protein interaction clusters and major functional groups.

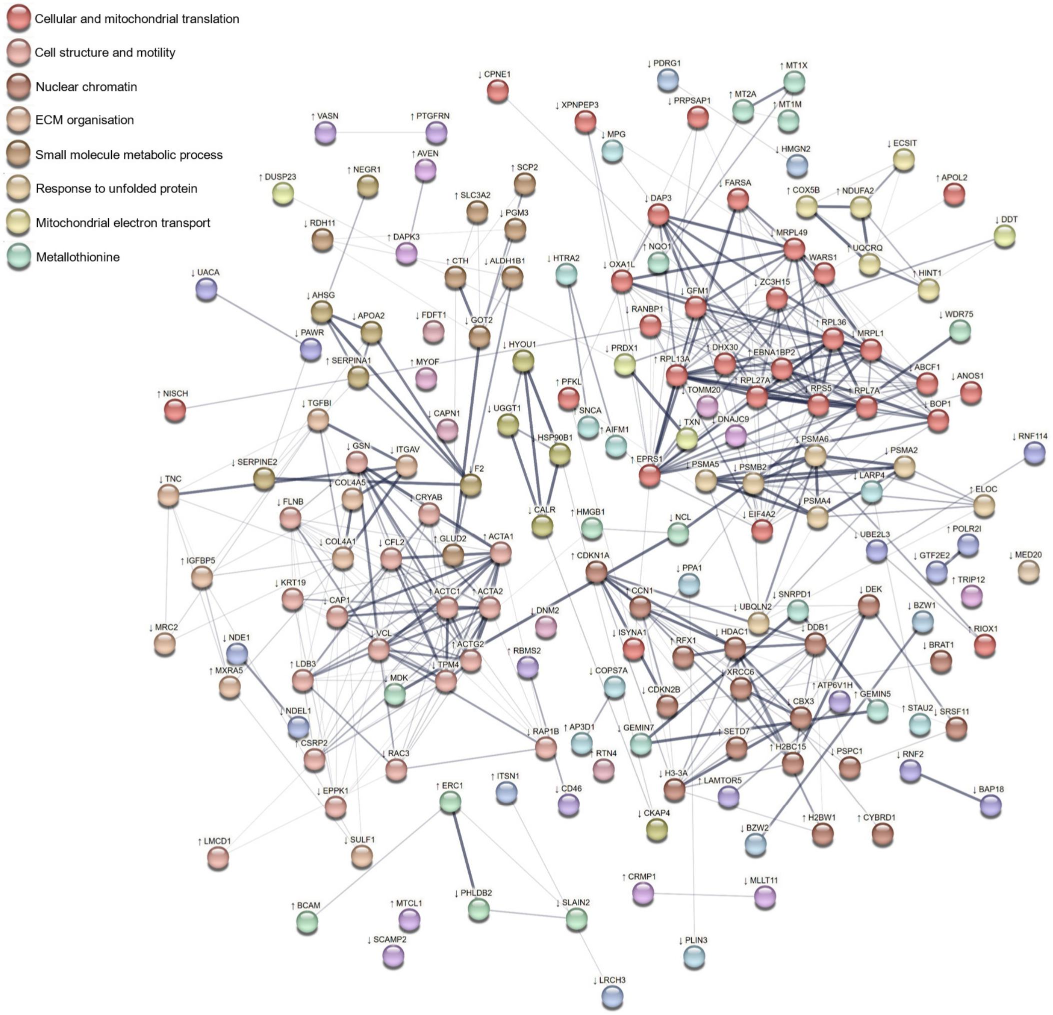

Figure S5. Differentially expressed edaravone-treated VWMD astrocyte protein-protein interaction clusters and major functional groups.

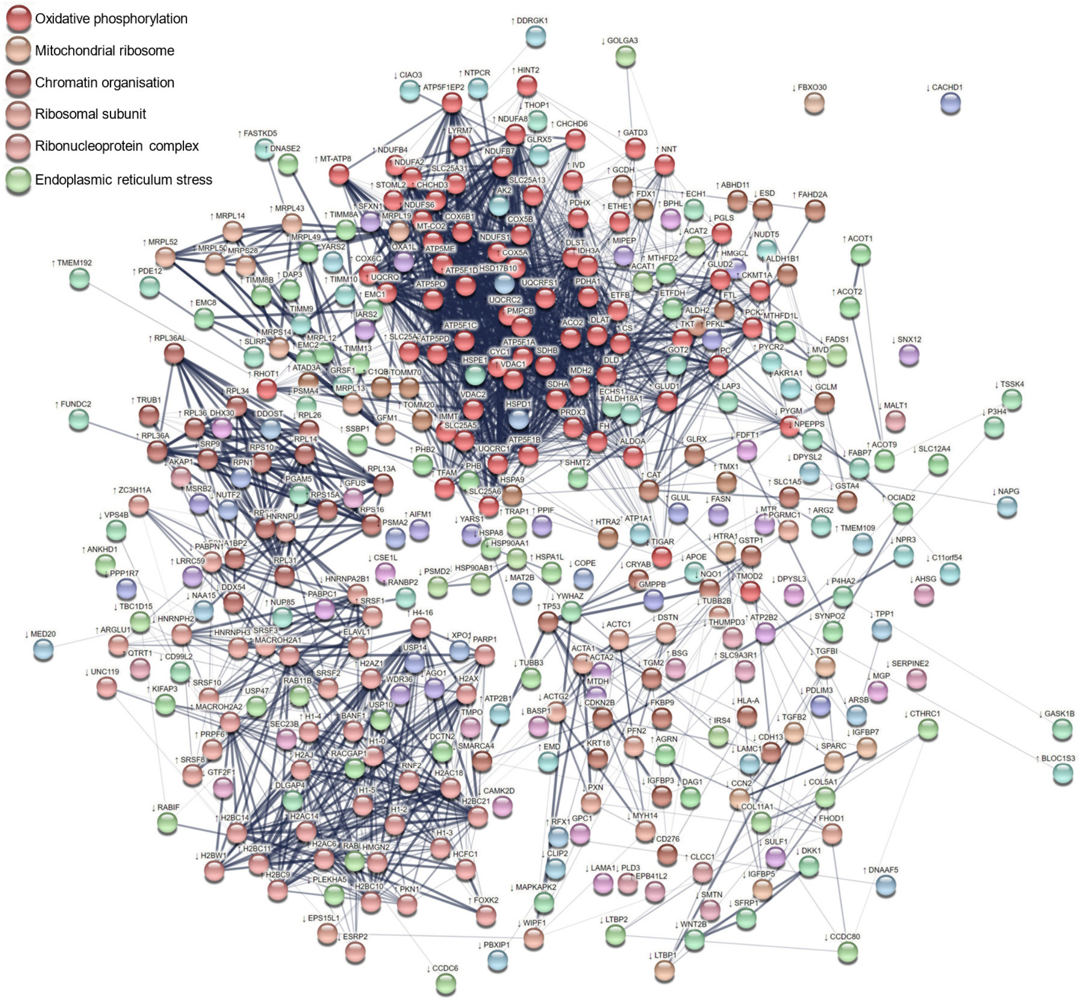

Figure S6. Differentially expressed VWMD astrocyte with mitochondrial transfer treatment protein-protein interaction clusters and major functional groups.

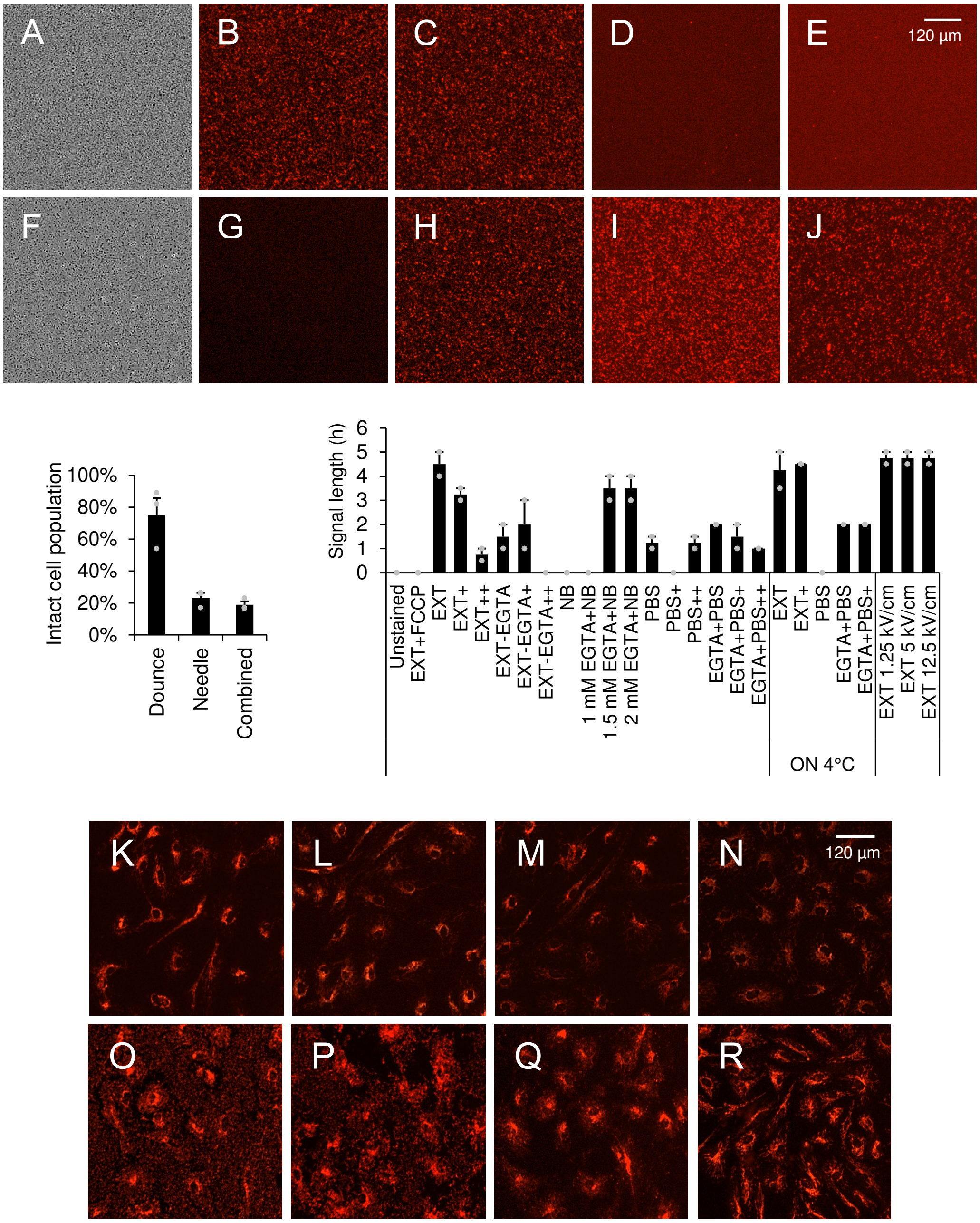

Figure S7. Mitochondrial extraction and buffer optimisation. Mitochondria were extracted by dounce homogenisation (30 strokes) followed by trituration by needle (25G, 3 triturations), after comparison with dounce (30 strokes) or needle extraction (3 dounce strokes, 30 needle triturations) (n = 3). TMRE staining of mitochondria in extraction buffer (EXT) (A, B), or with 1 µM FCCP (F, G), following electroporation at 1.25 kV/cm (C) or 12.5 kV/cm (H), in neural medium (B27-based medium, NB) (D) with 1 mM EGTA (E), 1.5 mM EGTA (I) or 2 mM EGTA (J). Signal length of TMRE staining in buffer conditions with extraction buffer (EXT), PBS + 0.1% BSA, with 1 mM $MgCl_2$ (+), or 1 mM $MgCl_2$ and 1 mM $CaCl_2$ (++) in presence or absence of EGTA, including following overnight incubation in extraction buffer at 4˚C (ON) or following electroporation (n = 2). VWMD astrocytes incubated in presence (O-R) and absence (K-N) of 10 cell equivalents of mitochondrial extract with representative images upon addition (K, O), 1 h (L, P), 4 h (M, Q) and 24 h (N, R).

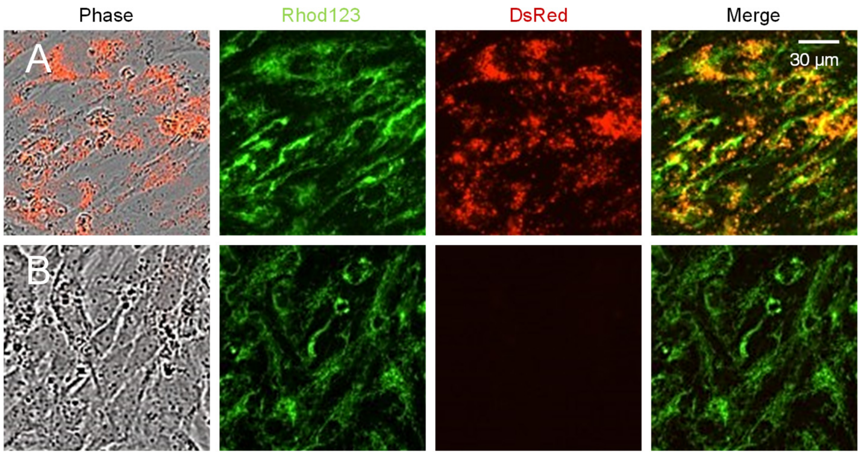

Figure S8. Cytoplasmic localisation of internalised pLV-mitoDsRed mitochondria in VWMD astrocytes. (A) V6 astrocytes treated with mitochondria tagged with pLV-mitoDsRed for 48 h, (B) untreated V6 astrocytes.

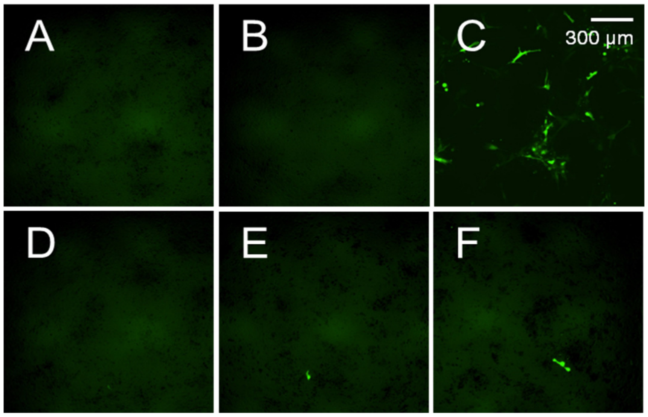

Figure S9. Mitochondria electroporation transfected with pEGFP-N1 into VWMD astrocytes. (A) NT, (B) eGFP, (C) eGFP with lipofectamine 3000, (D) mitochondria alone, (E) MT+eGFP 1.25 kV/cm, (F) MT+eGFP 12.5 kV/cm.

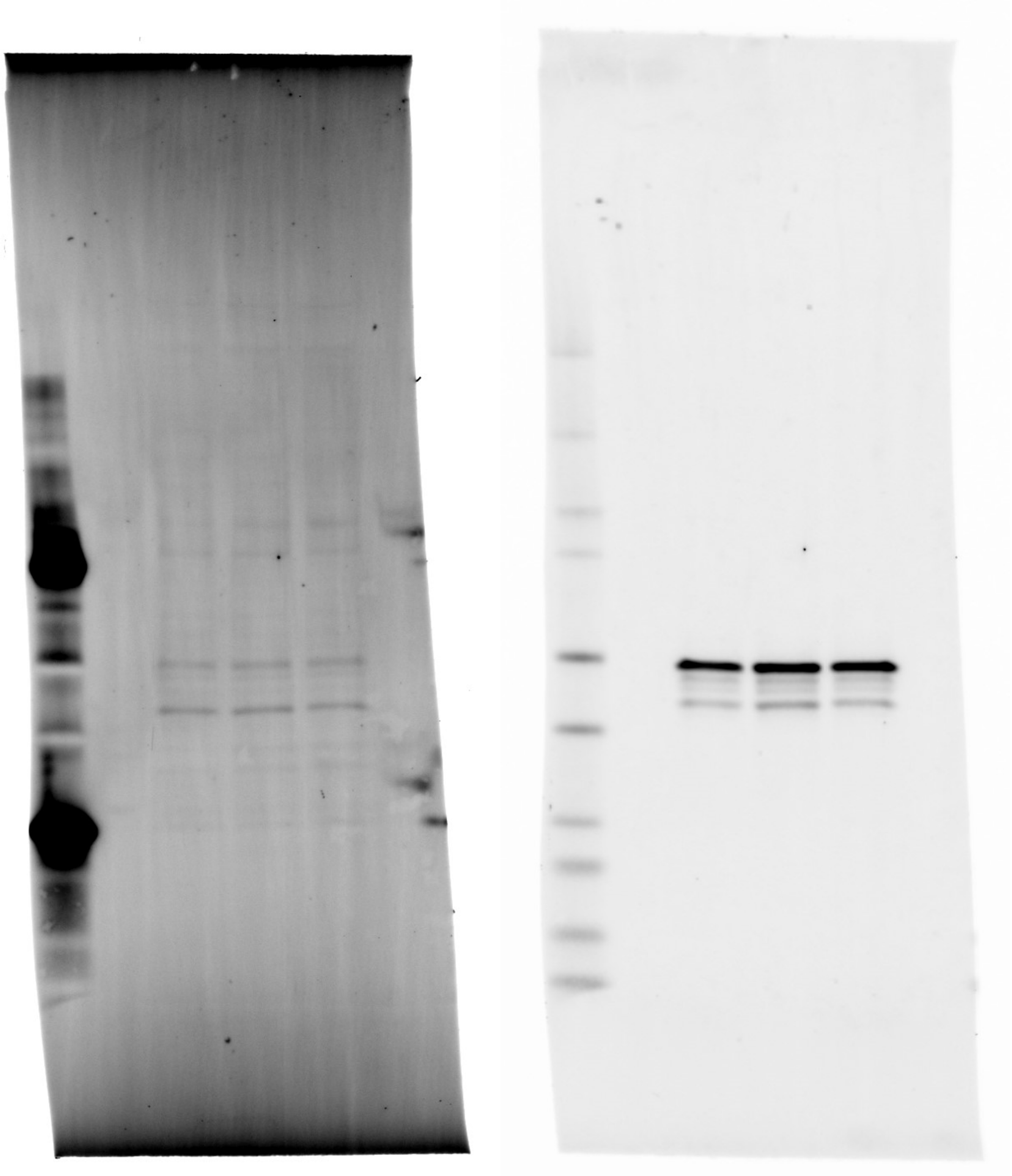

Figure S10. Western blot of edaravone and mitochondrial transfer treated VWMD astrocytes. Full unedited gel/blot for Figure 8. Lane 1 = control, Lane 2 = mitochondrial transfer, Lane 3 = edaravone. Stain-free blot (left) and GFAP stained blot (right).
